# Comprehensive metabolomics expands precision medicine for triple-negative breast cancer

**DOI:** 10.1038/s41422-022-00614-0

**Published:** 2022-02-01

**Authors:** Yi Xiao, Ding Ma, Yun-Song Yang, Fan Yang, Jia-Han Ding, Yue Gong, Lin Jiang, Li-Ping Ge, Song-Yang Wu, Qiang Yu, Qing Zhang, François Bertucci, Qiuzhuang Sun, Xin Hu, Da-Qiang Li, Zhi-Ming Shao, Yi-Zhou Jiang

**Affiliations:** 1 Key Laboratory of Breast Cancer in Shanghai, Department of Breast Surgery, Fudan University Shanghai Cancer Center; Department of Oncology, Shanghai Medical College, Fudan University, Shanghai, China; 2 Human Phenome Institute, Fudan University, Shanghai, China; 3 Department of Pathology, University of Texas Southwestern Medical Center, Dallas, TX USA; 4 Predictive Oncology team, Centre de Recherche en Cancérologie de Marseille (CRCM), INSERM UMR1068, CNRS UMR725, Aix-Marseille Université, Institut Paoli-Calmettes, Marseille, France; 5 Department of Industrial Systems Engineering and Management, National University of Singapore, Singapore, Singapore

**Keywords:** Breast cancer, Cancer metabolism

## Abstract

Metabolic reprogramming is a hallmark of cancer. However, systematic characterizations of metabolites in triple-negative breast cancer (TNBC) are still lacking. Our study profiled the polar metabolome and lipidome in 330 TNBC samples and 149 paired normal breast tissues to construct a large metabolomic atlas of TNBC. Combining with previously established transcriptomic and genomic data of the same cohort, we conducted a comprehensive analysis linking TNBC metabolome to genomics. Our study classified TNBCs into three distinct metabolomic subgroups: C1, characterized by the enrichment of ceramides and fatty acids; C2, featured with the upregulation of metabolites related to oxidation reaction and glycosyl transfer; and C3, having the lowest level of metabolic dysregulation. Based on this newly developed metabolomic dataset, we refined previous TNBC transcriptomic subtypes and identified some crucial subtype-specific metabolites as potential therapeutic targets. The transcriptomic luminal androgen receptor (LAR) subtype overlapped with metabolomic C1 subtype. Experiments on patient-derived organoid and xenograft models indicate that targeting sphingosine-1-phosphate (S1P), an intermediate of the ceramide pathway, is a promising therapy for LAR tumors. Moreover, the transcriptomic basal-like immune-suppressed (BLIS) subtype contained two prognostic metabolomic subgroups (C2 and C3), which could be distinguished through machine-learning methods. We show that N-acetyl-aspartyl-glutamate is a crucial tumor-promoting metabolite and potential therapeutic target for high-risk BLIS tumors. Together, our study reveals the clinical significance of TNBC metabolomics, which can not only optimize the transcriptomic subtyping system, but also suggest novel therapeutic targets. This metabolomic dataset can serve as a useful public resource to promote precision treatment of TNBC.

## Introduction

Triple-negative breast cancer (TNBC) is a subset of breast cancer defined by the lack of expression of estrogen receptor, progesterone receptor and human epidermal growth factor receptor 2.^1^ Clinical management of TNBC is a great challenge because of its high incidence of visceral metastases and the lack of well-recognized therapeutic targets.^1^ TNBC has been considered as a highly heterogeneous disease.^2–4^ Our previous study presented a genomic and transcriptomic landscape of 465 Chinese patients with TNBC and classified TNBCs into four transcriptomic subtypes with distinct molecular features.^4^ We further revealed the potential therapeutic targets of each transcriptomic subtype and conducted an umbrella trial (FUTURE, ClinicalTrials.gov, number: NCT03805399) on metastatic TNBCs to evaluate the treatment efficacy regarding to these targets.^5^ The FUTURE trial exhibited an encouraging objective response rate (ORR) of 29%, which was significantly higher than the ORRs of traditional chemotherapies (5%–15%).^6,7^ However, the treatment outcomes of corresponding targeted therapies did not reach our full expectation, especially for transcriptomic basal-like immune-suppressed (BLIS) and luminal androgen receptor (LAR) tumors. For example, CDK4/6, androgen receptor and mTOR inhibitors did not perform as well as expected in LAR tumors. Therefore, we need to seek for a more multilayered understanding of TNBC for new target identification.

As an important hallmark of cancer, metabolic reprogramming in TNBC is worthy of further exploration. Previously, the mRNA expression level of metabolic genes were usually interpreted as the activity of corresponding metabolic pathways.^8,9^ Our recent study used the transcriptomic data of metabolic genes to investigate the metabolic features of TNBC.^9^ This study revealed that TNBCs could be classified into three metabolic-gene-based subtypes with distinct dependency on lipid metabolism and glycolysis. We further proposed therapeutic strategies targeting classic energy metabolism and developed combination therapies using glycolysis inhibitors together with immune checkpoint inhibitors.^9^ However, metabolic research using transcriptomic data still has limitations. First, the regulation of metabolism is a complex process. The abundance of metabolites might be more reliable for metabolic flux analysis than the mRNA expression of metabolic genes. Second, some crucial metabolites that are not in the classical energy metabolism pathways might be ignored, as previous transcriptomic data-based metabolic studies mainly focused on energy metabolism. Therefore, a more direct illustration of metabolism through analyzing the abundance of metabolites is required.

**Fig. 1:**
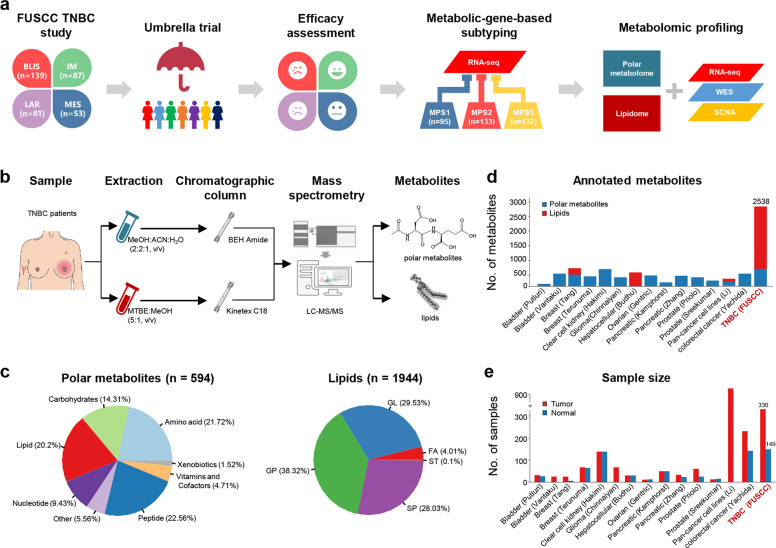
Overview of polar metabolome and lipidome detection in TNBC. **a** A sketch map showing the combined analysis using previously obtained transcriptomic data and the metabolomics data reported in this study for TNBC precision medicine. **b** A schematic summarizing the workflow for metabolite profiling. **c** The numbers and proportions of annotated polar metabolites and lipids in our study. Comparison of the number of annotated metabolites (**d**) and the number of samples (**e**) between our study and previous studies. BLIS, basal-like immunesuppressed; IM, immunomodulatory; LAR, luminal androgen receptor; MES, mesenchymal-like; MPS, metabolic-pathway-based subtypes; FA, fatty acids; GL, glycerolipids; GP, glycerophospholipids; SP, sphingolipids; ST, sterol lipids.

The rapid development of high-throughput metabolomics techniques, such as global untargeted metabolomics and lipidomics, has paved the way for directly measuring the abundance of metabolites.^10,11^ Using these methods, we have achieved a deeper understanding of metabolic features in breast cancer.^12–14^ Previous studies demonstrated significant differences in glutamine and alanine metabolism between ER-positive and ER-negative breast cancers.^12,13^ Besides, the *MYC*-driven accumulation of 2-hydroxyglutarate has been shown to promote breast cancer progression.^14^ However, large-scale metabolomic researches connecting metabolism to TNBC genomics are still lacking. In this study, we profiled both the polar metabolome and lipidome of 330 TNBC samples to reveal the metabolomic landscape. We also integrated the metabolomics with previously established genomic and transcriptomic data to explore the potential metabolic targets for TNBCs, especially for transcriptomic BLIS and LAR tumors (Fig. 1a).

## Results

### Polar metabolite and lipid profiling of TNBC

To comprehensively profile the TNBC metabolome, we used the sample set of our TNBC cohort containing sufficient quantities of high-quality fresh frozen tissues for polar metabolome and lipidome detection (Fig. 1b). Our metabolomic cohort included 330 TNBC samples and 149 paired normal breast tissues. We annotated a total of 594 polar metabolites and 1944 lipids (Fig. 1c; Supplementary information, Tables S1–S4). The data quality was checked with internal standards and quality control samples (Supplementary information, Data S1 and Fig. S1).

We previously obtained transcriptomic data for 258 out of these 330 samples, among which 171 samples have whole-exome sequencing (WES) and somatic copy number alteration (SCNA) data as well (Supplementary information, Fig. S2). Generally, the newly developed TNBC metabolomic dataset is a large data resource regarding both the number of annotated metabolites and sample size (Fig. 1d, e).^14–27^

### The metabolomic landscape of TNBC

**Fig. 2:**
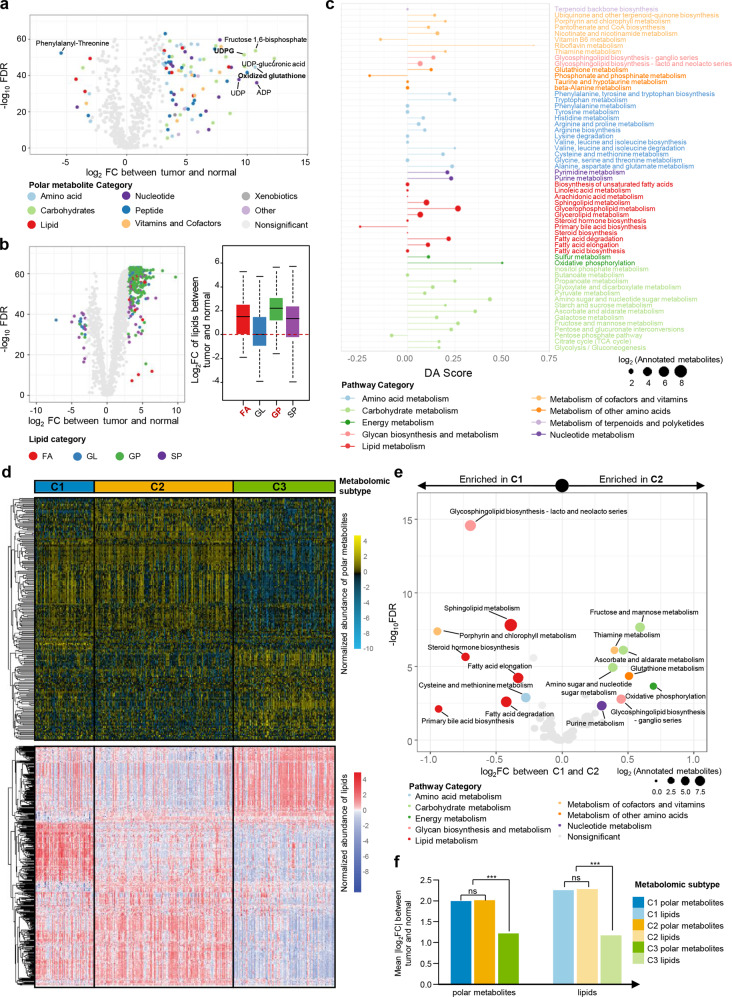
The metabolomic landscape of triple-negative breast cancer. **a**, **b** volcano plots of the 594 annotated polar metabolites (**a**) and 1944 lipids (**b**) profiled. Metabolites of different categories were individually color-coded. Right part of panel **b**: Log_2_ fold changes of the abundances of different categories of lipids in TNBC tumor tissues as compared with normal tissues. Log_2_ fold change value of 0 (the dashed red line) indicates the same level of lipid abundance between the tumor and the normal. **c** A pathway-based analysis of metabolomic changes between tumor and normal tissues. The DA score captures the average, gross changes for all metabolites in a pathway. A score of 1 indicates that all measured metabolites in the pathway increase in the tumor compared to normal tissues, and a score of −1 indicates that all measured metabolites in a pathway decrease. Pathways with no less than three measured metabolites were used for DA score calculation. **d** SNF clustering of metabolomic data. **e** Pathway abundance (PA) scores between C1 and C2 subtypes. The PA score was calculated as the mean log_2_ fold change of the abundances of measured metabolites in this pathway. **f** Degree of overall metabolomic dysregulation among three metabolomic subtypes. For each metabolomic subtype, the mean log_2_ fold change of metabolites between tumor and normal tissues was calculated to represent the overall degree of metabolomic dysregulation.

Using Benjamini–Hochberg-corrected Mann–Whitney *U* tests, we identified 452 metabolites (417 higher and 35 lower in tumor) displaying significant differences in abundance between tumor and normal samples (Fig. 2a, b). Among the dysregulated metabolites, some polar metabolites, especially metabolites related to oxidation reaction and glycosyl transfer (such as oxidized glutathione [GSSG] and uridine diphosphate glucose [UDPG]), were significantly enriched in tumors compared with normal tissues (Fig. 2a). Some lipids, such as phosphatidylinositols, fatty acids (FAs) and ceramides, were also enriched in TNBCs (Fig. 2b; Supplementary information, Fig. S3a). Moreover, we conducted the Spearman’s correlation analysis which illustrated that lipids belonging to the same subclass were closely correlated based on their abundances, we thus considered lipids of the same subclass as a whole for subsequent lipidomic analysis (Supplementary information, Fig. S3b). Furthermore, we performed KEGG metabolic pathway-based differential abundance (DA) analysis between tumor and normal tissues to investigate the dysregulation of metabolic pathways^24^ (Fig. 2c; Supplementary information, Fig. S3c). In our research, a large number of metabolites involved in glycerophospholipid metabolism, amino sugar and nucleotide sugar metabolism pathways showed high DA scores.

Then, we applied the similarity network fusion (SNF)^28^ method to explore the intertumoral metabolomic heterogeneity of TNBCs. TNBCs could be clearly divided into three subgroups by this analysis (Fig. 2d; Supplementary information, Fig. S4a and Table S5). We further validated the robustness of the clustering results by using different numbers of metabolites for analysis and by choosing different clustering methods (Supplementary information, Fig. S4b–d). The metabolomic C1 subtype was featured with sphingolipids and FAs enrichments, while the metabolomic C2 subgroup was characterized by upregulated carbohydrate metabolism and oxidation reaction (Fig. 2e; Supplementary information, Fig. S5a, b). The metabolomic C3 subgroup showed mild metabolic differences compared with normal tissue (Fig. 2f). In terms of energy metabolism, metabolomic C1 tumors showed enriched long-chain and unsaturated FA and might be more dependent on fatty acid metabolism; metabolomic C2 tumors were relatively more abundant with metabolites in glutamate pathways and might rely more on glutamate metabolism; metabolomic C3 tumors had smaller metabolomic difference as compared with normal tissues (Supplementary information, Fig. S5c). The three metabolomic subtypes exhibited no difference in cancer cell fractions, and they had similar clinicopathological features except that patients in C1 were older in age at diagnosis (Supplementary information, Fig. S5d and Table S6). Generally, the metabolomic clustering of TNBCs revealed metabolic heterogeneity and provided insight for further target exploration.

### A comprehensive analysis linking polar metabolites and lipids to genomic features

**Fig. 3:**
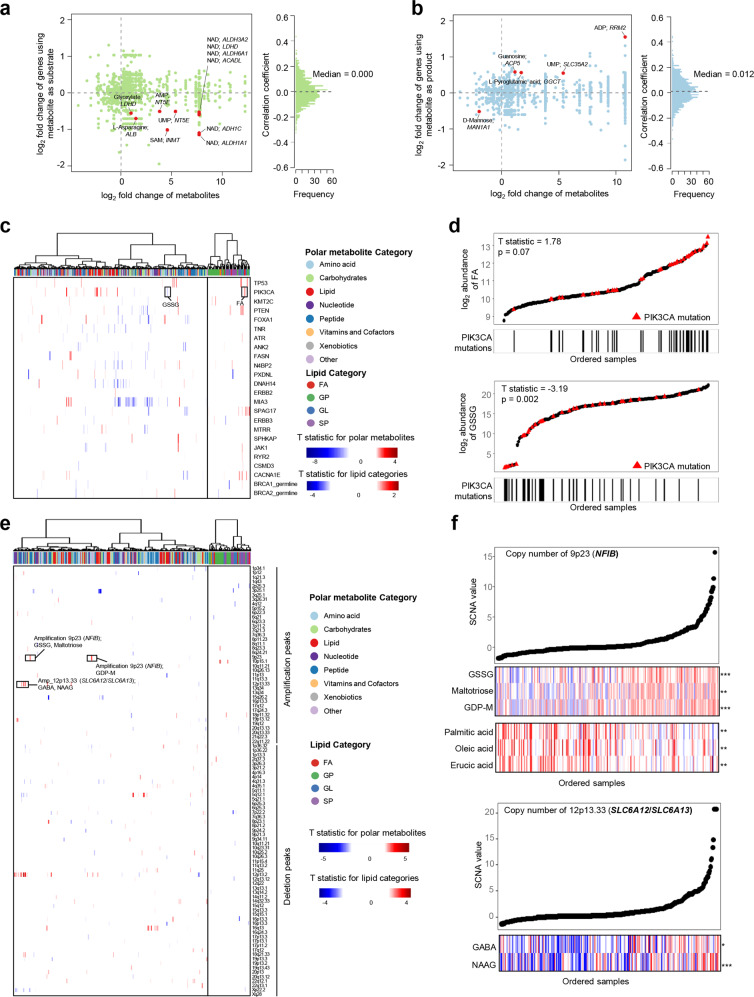
Systematic evaluations linking polar metabolome and lipidome to genomic features. **a**, **b** Correlation of mRNA expression of metabolic genes with the abundances of paired metabolites as substrates (**a**) or products (**b**). Metabolite-gene pairs were derived from the Recon 3D dataset. Pairs with significant differences between tumor and normal tissues and significant correlations were annotated in the plot. **c** Heatmap showing the associations between the abundances of metabolites and the presence of mutations within the indicated genes. The mutations include high frequency somatic mutations (mutated in at least 6% of the cases in at least one metabolomic subtype) within cancer-related genes and high frequency germline mutations in *BRCA1* and *BRCA2*. T statistics were calculated by a linear regression model that adjusted the cofounding factors. **d** Correlations between *PIK3CA* mutations and FA subclass (top panel) and GSSG levels (bottom panel). All lipids belonging to the FA subclass (*n* = 10) were included. The mean abundance of the ten metabolites was regarded as the abundance of FA subclass. All samples were ordered based on the abundance (*y*-axis) of FA subclass (top panel) or GSSG (bottom panel), and the ones with *PIK3CA* mutations were highlighted in red and indicated by the corresponding lines displayed in *x*-axis. **e** Heatmap showing the associations between abundances of metabolites and copy number values of TNBC SCNA peaks. T statistics were calculated by a linear regression model that adjusted the cofounding factors. **f** Top panel: correlations between the copy number values of 9p23 and the abundances of GSSG, maltotriose, GDP-M and some FAs. Bottom panel: correlations between the copy number values of 12p13.33 and the abundances of GABA and NAAG. SCNA-related metabolites are shown as lines and samples were ordered by increasing copy number values. The abundances of the metabolite are illustrated in colors. ***P < 0.001, **P < 0.01; *P < 0.05; ns, *P* ≥ 0.05.

We also explored the associations between metabolomics and genomic features to speculate on the potential genomic drivers contributing to the formation of metabolomic features. The correlations between the polar metabolite abundance and the mRNA expression of paired metabolic genes were analyzed based on the Recon3D database.^29^ Generally, these pairs illustrated weak correlations, reflecting the complexity of the metabolic network (Fig. 3a, b; Supplementary information, Tables S7 and S8). A few metabolites and their paired metabolic genes, such as D-mannose and *MAN1A1*, had significant correlations (Supplementary information, Fig. S6a).

Furthermore, we focused on a list of 24 known cancer-related genes that are frequently mutated in TNBCs^30,31^ and applied a linear regression model (controlling the cofounding factors) to investigate the associations between somatic mutations and metabolite abundance (Fig. 3c; Supplementary information, Table S9). Although *TP53* mutations, the most prominent cancer-related alterations in TNBC, generally had weak associations with the profiled metabolites, we found that *PIK3CA* mutations were positively associated with the abundance of FA but negatively correlated with that of GSSG (Fig. 3d; Supplementary information, Fig. S6b). Consistently, the overproduction of FA (especially arachidonic acid) driven by *PIK3CA* mutation has recently been reported.^32^ Moreover, we also analyzed the correlation of the mRNA expression of breast cancer-related genes^28^ with metabolites (Supplementary information, Fig. S6c and Table S10). For example, we observed the positive correlation of *BUB1B* mRNA expression with S-adenosylmethionine abundance (Supplementary information, Fig. S6d).

In terms of copy number, the associations between SCNAs and metabolites were generally not strong, and we only identified a few TNBC-specific SCNA peaks^4^ related to metabolite abundance (Fig. 3e; Supplementary information, Table S11). For example, the copy number of the 9p23 chromosomal region, within which the oncogenic gene *NFIB* locates, was positively correlated with the abundances of GSSG, maltotriose and guanosine diphosphate mannose (GDP-M) but negatively correlated with the abundances of a few FAs (Fig. 3f). Besides, we investigated the chromosomal region 12p13.33 that includes genes encoding *SLC6A12*/*SLC6A13*, which function as neurotransmitter transporters in the membrane. Our study showed that the copy number of the 12p13.33 chromosomal region was positively correlated with the abundances of the neurotransmitters gamma-aminobutyric acid (GABA) and N-acetyl-aspartyl-glutamate (NAAG) (Fig. 3f). In summary, the analysis of the associations between genomic features and metabolites might provide hints for the driving forces of metabolic reprogramming in TNBC.

### Metabolomic subtyping refines the transcriptomic subtyping of BLIS tumors and can be achieved by machine learning

**Fig. 4:**
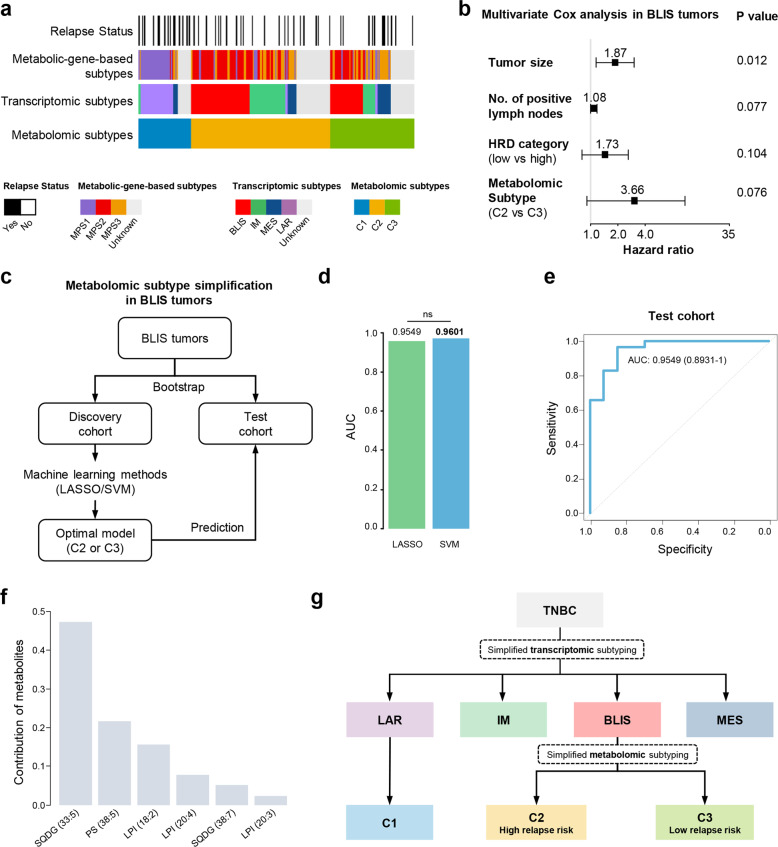
Metabolomic subtyping refines the transcriptomic subtyping in BLIS tumors and can be achieved by machine-learning methods. **a** Associations of metabolomic subtypes with transcriptomic subtypes, metabolic-gene-based subtypes and relapse status of TNBCs. **b** Association of tumor size, number of positive lymph nodes, homologous recombination defect (HRD) categories and metabolomic subtypes with relapse-free survival (RFS) in patients with BLIS tumors. Multivariate Cox regression model was used for analysis. The hazard ratios were shown with 95% confidence intervals. Proportion hazard assumption was tested in advance. **c** Design of the analytical pipeline for metabolomic subtyping via machine-learning methods for patients with BLIS tumors. Bootstrap method was used for the classification of discovery and test cohorts. Two machine-learning methods (LASSO and SVM) were used for model construction. **d** Comparison of the efficacies of two machine-learning methods for the test cohort. **e** Efficacy of the LASSO regression model in predicting metabolomic subtypes of BLIS tumors was reflected by ROC curves with AUCs reported. **f** Contribution of the six metabolites to the LASSO regression model. **g** The integration of transcriptomic and metabolomic subtyping system for potential clinical utilization. The simplified transcriptomic subtyping through four immunohistochemistry markers was previously developed by our group and widely used in clinical setting in our center. LASSO, the least absolute shrinkage and selectionator operator; SVM, Support Vector Machine; SQDG, sulfoquinovosyl diacylglycerol; LPI, Lysophosphatidylinositol; PS, phosphatidylserine.

We further explored the associations among metabolomic subtypes, previously defined transcriptomic subtypes^4^ and metabolic-pathway-based subtypes (MPSs).^9^ In terms of transcriptomic subtypes, the LAR subtype almost overlapped with the metabolomic C1 subtype; and the BLIS, immunomodulatory (IM) and mesenchymal-like (MES) subtypes were primarily divided into metabolomic C2 and C3 subtypes. In regard to MPSs, the MPS1 subtype was highly consistent with the metabolomic C1 subtype, while the MPS2 and MPS3 subtypes were interlaced with the metabolomic C2 and C3 subtypes (Fig. 4a).

We also explored the prognostic value of metabolomic subtypes and demonstrated that BLIS tumors contained two prognostic metabolomic subgroups (C2 and C3). For BLIS tumors, metabolomic C2 subtype had worse relapse-free survival (RFS) compared with metabolomic C3 subtype (Supplementary information, Fig. S7a, b). After adjusting for tumor size, number of positive lymph nodes and homologous recombination defect score categories, the metabolomic subtype still tended to be an independent prognostic factor (Fig. 4b) for BLIS patients. We next tried to build a simplified metabolomic subtyping system for BLIS tumors by machine-learning methods. The bootstrap method was utilized to develop the discovery and test cohorts. For the discovery cohort, we developed the working model using two machine-learning methods, the least absolute shrinkage and selection operator (LASSO) and support vector machine (SVM). The established model was then run in the test cohort (Fig. 4c). The LASSO and SVM methods both had favorable predictive efficacy in the test cohort (Fig. 4d). As the LASSO model can directly show the included metabolites and their contributions, we further demonstrated the predictive efficacy of the LASSO model by receiver operating characteristic (ROC) curve as well as the contributions of the six metabolites included (Fig. 4e, f; Supplementary information, Fig. S7c). These data illustrated that machine-learning methods successfully distinguished the two metabolomic subtypes within BLIS tumors.

To prepare for cell line-based experiments, we also conducted transcriptomic-based and metabolite-based subtyping for TNBC cell lines (Supplementary information, Fig. S8a–c). MDA-MB-453 and MFM-223 cell lines were classified into the metabolomic C1 (transcriptomic LAR) subtype; HCC1806, HS-578T and LM2-4175 cell lines might be candidates for the metabolomic C2 (transcriptomic non-LAR) subtype. Similar to the findings for TNBC samples, cell lines of metabolomic C1 subtype were more dependent on fatty acids while cell lines of metabolomic C2 subtype were more dependent on glutamine as mitochondrial fuel (Supplementary information, Fig. S8d).

In summary, metabolomic profiling refines the previously developed transcriptomic subtypes of TNBC. Considering the unsatisfactory treatment efficacy for LAR and BLIS patients in our FUTURE trial,^5^ the integration of metabolomic subtyping with transcriptomic subtyping should be considered for further investigation (Fig. 4g). For LAR tumors, further exploration of metabolic targets could be conducted based on the features of the metabolomic C1 subtype. For BLIS patients, the simplified machine-learning-based metabolomic subtyping system could potentially stratify them into groups with distinct recurrence risks.

### Analysis of ceramide metabolism in the LAR subtype revealed sphingosine-1-phosphate (S1P) as a potential therapeutic target

**Fig. 5:**
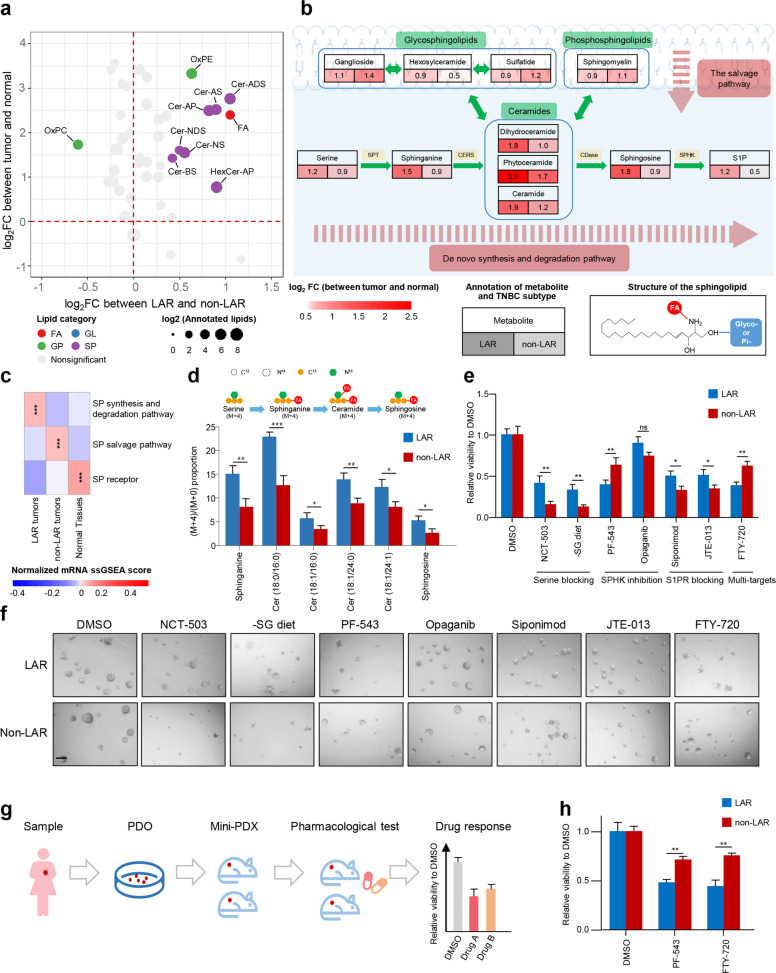
Analysis of ceramide metabolism in the LAR subtype revealed S1P as a potential therapeutic target. **a** Log_2_ fold change of lipid subclasses between tumor and normal tissues and between LAR and non-LAR tumors. Log_2_ fold change of each lipid subclass was calculated as the mean log_2_ fold change of the abundances of lipids belonging to this subclass. **b** Metabolomic changes in sphingolipid (SP) metabolism. Log_2_ fold changes of the abundances of metabolites in tumor samples (LAR or non-LAR) as compared with normal tissues were illustrated. **c** Transcriptomic changes in three SP metabolism-related pathways. ssGSEA scores of the pathways based on transcriptomics were calculated and compared among LAR tumors, non-LAR tumors and normal tissues. **d** Proportions of isotope-labeled intermediates that are involved in the de novo synthesis and degradation of the ceramide pathway in LAR and non-LAR cell lines. MDA-MB-453 and MFM-223 cell lines of LAR subtype as well as BT-549 and LM2-4175 cell lines of non-LAR subtype were used for experiments. Each sample was detected with three replicates. **e**, **f** Viability detection of PDOs after blocking different steps involved in de novo synthesis and degradation of ceramide pathway (*n* = 5 different PDOs with three replicates for each group). The efficacy of inhibition (**e**) and representative images (**f**) were illustrated. The concentrations of the inhibitors were as follows: NCT-503, 30 µM; PF-543, 10 µM; Opaganib, 50 µM; Siponimod, 30 µM; JTE-013, 30 µM; FTY-720, 1 µM. **g** Pharmacological tests of PF-543 and FTY-720 using mini-PDX models. **h** Drug sensitivity results for mini-PDX models of LAR and non-LAR tumors (*n* = 3 different mini-PDX with three replicates for each group). Statistical comparisons in **d**, **e** and **h** were conducted using two-tailed Student’s *t-*test. Data are presented as means ± SEM. Scale bars, 200 μm. ***P < 0.001, **P < 0.01; *P < 0.05; ns, *P* ≥ 0.05. Cer, ceramides; AS, α-hydroxy fatty acid-sphingosine; AP, α-hydroxy fatty acid-phytospingosine; NS, non-hydroxyfatty acid-sphingosine; BS, β-hydroxy fatty acidsphingosine; ADS, α-hydroxy fatty acid-dihydrosphingosine; NDS, non-hydroxy fatty acid-dihydrosphingosine; HexCer, Hexosylceramide; OxPE, oxidized phosphatidylethanolamine; OxPC, oxidized phosphatidylcholine; SPT, serine palmitoyltransferases; CERS, (dihydro)ceramide synthases; CDase, ceramidase; SPHK, sphingosine kinase.

Transcriptomic LAR subtype almost overlapped with the metabolomic C1 subtype (Fig. 4a) that was featured by enrichment of sphingolipid metabolism-related metabolites (Fig. 2e). Therefore, we analyzed detailed intermediates of the sphingolipid metabolism pathway to identify crucial metabolites for LAR tumors. In comparison with the normal tissues and the non-LAR tumors, LAR tumors were characterized by the enrichment of ceramides (Fig. 5a). Further pathway analysis of metabolites and mRNA expression of related metabolic genes showed that de novo synthesis and degradation of ceramide pathway was more active in LAR tumors, whereas the transfer of glycosyl and phosphate groups was not significantly upregulated compared with non-LAR tumors (Fig. 5b, c). These results suggest the crucial role of the de novo synthesis and degradation of ceramide pathway in LAR tumors. In particular, we conducted the targeted detection of the crucial intermediates in this pathway, including sphinganine, sphingosine and S1P, to validate their enrichments in LAR tumors (Supplementary information, Fig. S9a). Moreover, we also utilized stable isotope tracing experiments to illustrate the active de novo synthesis and degradation of ceramide pathway in the LAR subtype (Fig. 5d).

To investigate potential therapeutic strategies for LAR tumors, we systematically blocked each step of de novo synthesis and degradation of ceramide pathway in cell lines and patient-derived organoid (PDO) models. The transcriptomic subtype of the PDO was defined by the immunohistochemistry method (see Materials and Methods).^33^ As illustrated in Fig. 5e, f and Supplementary information, Fig. S9b, PF-543 (an inhibitor of SPHK1) and FTY-720 (also known as fingolimod, an FDA-approved multi-target drug of the ceramide pathway^34,35^) were significantly more effective on LAR tumors. Meanwhile, NCT-503 or serine deprived medium that blocks serine obtaining, Fumonisin B1 that inhibits (dihydro)ceramide synthases (CERS), Opaganib that inhibits sphingosine kinase 2 (SPHK2) or Siponimod and JTE-013 that block S1P-S1P receptors (S1PRs) binding were more effective on non-LAR tumors or were ineffective in both LAR and non-LAR tumors. These results revealed the importance of SPHK1 and the tumor-promoting metabolite S1P in LAR tumors. We also validated the on-target efficacy of SPHK1 inhibitors (PF-543 and SK-IN-1) by quantifying the decrease of S1P after the utilization of SPHK1 inhibitors. The on-target efficacy was further confirmed by the observation that the efficacy of SPHK1 inhibitors decreased upon knockdown of *SPHK1* (Supplementary information, Fig. S9c–e). Furthermore, we tested the efficacy of PF-543 and FTY-720 using mini patient-derived xenograft (mini-PDX) models (Fig. 5g). Consistent with the results in the PDO models, mini-PDX models of the LAR subtype were more sensitive to the treatment of PF-543 and FTY-720 (Fig. 5h).

In conclusion, our results suggest S1P, an important intermediate of ceramide pathway, played a crucial role in LAR tumors. PF-543 and FTY-720 might be the subtype-specific therapies for LAR tumors.

### Identification of NAAG as a crucial tumor-promoting metabolite in BLIS tumors

**Fig. 6:**
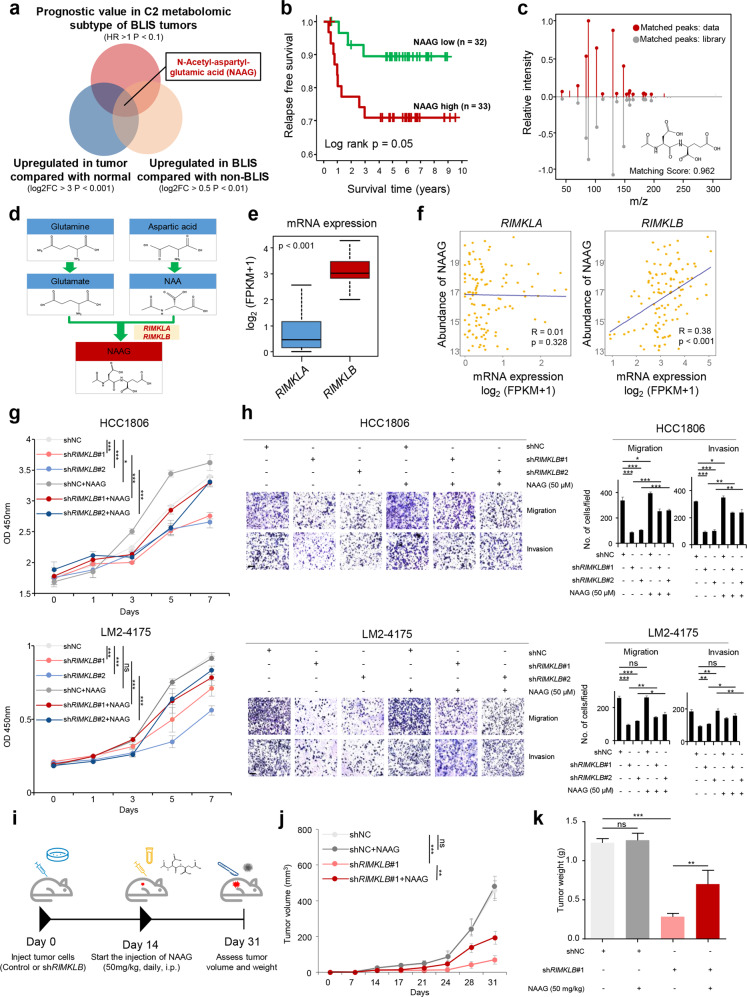
Identification of NAAG as a crucial tumor-promoting metabolite in BLIS tumors. **a** Screening criteria of metabolites potentially promoting tumor progression in BLIS tumors. **b** RFS of patients with different NAAG abundances of BLIS tumors. The *P* value was calculated by the log rank test. **c** Confirmation of NAAG by comparison with standard compound. Measured MS/MS spectral fragmentation profiles (top, in red) matched those of chemical standards (bottom, in gray). **d** Illustration of the NAAG metabolism pathway. **e**, **f** mRNA expression of *RIMKLA* and *RIMKLB* (**e**) and their relationship with NAAG abundance in BLIS tumors (**f**). **g** Quantification of cell proliferation after knocking down *RIMKLB* with shRNA and the complement of NAAG. **h** Right panel: quantification of cells migrating across transwell filters and invading through matrigel-coated transwells after knocking down *RIMKLB* with shRNA and the complement of NAAG. Left panel: representative images of three replicates. Ten random fields were counted per insert at 20×**. i** Experimental design. **j**, **k** Effect of *RIMKLB* knockdown and NAAG complement on tumor growth (**j**) and tumor weight (**k**) (*n* = 6 for each group). Statistical comparisons in **g**–**k** were conducted using two-tailed Student’s *t*-test. Data are presented as means ± SEM. Scale bars, 200 μm. ***P < 0.001, **P < 0.01; *P < 0.05; ns, *P* ≥ 0.05. NAA, N-acetyl-aspartic acid.

We further explored important tumor-promoting metabolites for BLIS tumors. After analyzing metabolites that were specifically upregulated and predicted poor prognosis in BLIS tumors, we identified NAAG as a potential candidate (Fig. 6a, b). We further validated the identity of this metabolite by chemical standard (Fig. 6c). Two enzymes, RIMKLA and RIMKLB, are reported to be responsible for the production of NAAG^36^ (Fig. 6d). As *RIMKLB* was significantly more abundant than *RIMKLA* at the transcriptomic level and positively correlated with the abundance of NAAG, we speculated that RIMKLB was the key enzyme determining the level of NAAG in TNBC (Fig. 6e, f). We first validated the positive correlation of mRNA expression of *RIMKLB* with NAAG abundance in TNBC cell lines. HCC1806 and LM2-4175 cell lines with relatively high *RIMKLB* expression and Hs-578T cell line with relatively low *RIMKLB* expression were chosen for further experiments (Supplementary information, Fig. S10a). When knocking down *RIMKLB* with shRNA in HCC1806 and LM2-4175 cell lines, we observed a significant decrease in NAAG abundance, accompanying with decreases in growth rates and abilities of migration and invasion. After we supplied 50 µM NAAG into the medium, the tumor inhibitory effect of *RIMKLB* depletion was partially rescued (Fig. 6g, h; Supplementary information, Fig. S10b, c). In addition, NAAG promoted the migration of Hs-587T cells and 50 µM was the optimal concentration based on our concentration gradient experiment (Supplementary information, Fig. S10d–f). The effects of *RIMKLB* and NAAG were further validated in vivo. Tumor growth was significantly decreased with the knockdown of *RIMKLB* and was partly rescued with NAAG supplementation (Fig. 6i–k; Supplementary information, Fig. S10g, h). These data demonstrated that NAAG is a crucial tumor-promoting metabolite in BLIS tumors, and targeting the biosynthesis of NAAG might be a feasible treatment strategy.

## Discussion

Metabolic reprogramming is a crucial hallmark of tumor and provides potential therapeutic targets. In this study, we constructed a large metabolomic dataset to systematically describe the metabolomic landscape of TNBC. We also demonstrated that the three TNBC metabolomic subtypes refined our previously defined TNBC transcriptomic subtypes (Fig. 4g). Furthermore, targeting the biosynthesis of some functional metabolites, such as S1P and NAAG, could be potentially effective for treatment of LAR and BLIS tumors, respectively. Overall, our study illustrated the metabolomic landscape and might expand precision medicine for TNBC.

With this newly constructed metabolomic dataset, we paved the TNBC metabolic research one step further. Our group previously utilized the transcriptomic data of metabolic genes to reveal TNBC metabolic features. The heterogeneity of energy metabolism was illustrated with potential therapeutic options.^9^ In this study, we explored the metabolic features more directly with the polar metabolome and lipidome. We revealed that metabolomic dysfunction in TNBC was characterized by an overall increase in the abundance of metabolites, different from the observations for tumors of other histological types.^37^ For example, clear cell renal cell carcinoma demonstrated balanced upregulated and downregulated metabolites (170 upregulated and 149 downregulated).^24^ We also noticed that some featured metabolites, such as UDPG and GDP-mannose that are specific to glycosyl transfer, were significantly increased in TNBC. Accordingly, the deregulation of glycosylation has been reported as a feature of TNBC and significantly affected the biological outcome.^38^ With paired genomic and transcriptomic data, we systematically delineated the correlations between genomic alterations and metabolites to suggest potential genomic drivers of metabolism. Consistently, FA production driven by *PIK3CA* mutation has recently been reported.^32^ Some of the newly identified correlations between metabolomics and genomics, such as *NFIB* amplification and GSSG enrichment, need further investigation.

In our study, several observations relate to potential clinical translation. First, we illustrated the refinement of metabolomic subtypes to previously defined transcriptomic subtypes (Fig. 4g). The simplified metabolomic subtyping system for BLIS tumors has the potential for clinical application. Furthermore, metabolomics was utilized to identify novel therapeutic targets. S1P, a well-known tumor-promoting intermediate of the ceramide pathway, was enriched in LAR tumors. The production and function of S1P included four steps: the intake of serine, the transformation of serine into ceramides, the degradation of ceramides to S1P and S1P binding to S1PRs. Therefore, we conducted experiments on cell lines and PDO models to block each step. The low efficacy of targeting some steps in LAR tumors have been suggested in previous studies.^39,40^ For example, blocking SPT and CERS have been reported to be ineffective as they inhibit the formation of 1-deoxy(dihydro)ceramides and thus promote tumor growth.^39^ Besides, serine restriction, especially through PHGDH inhibition, has been proven to be more effective for basal-like rather than non-basal-like tumors,^40^ which was consistent with our results. Our study emphasized the importance of blocking the formation of S1P with SPHK1 inhibitors in LAR tumors. Moreover, we also identified NAAG as a crucial tumor-promoting metabolite in BLIS tumors. NAAG was previously recognized as a neurotransmitter that functions in the central neural system.^41^ Recently, NAAG was also reported to function as a circulating biomarker and a therapeutic target.^42,43^ Inhibiting NAAG transformation into glutamate could restrain the growth of lymphoma and ovarian serous adenocarcinoma models.^43^ Considering the similarity between BLIS tumors and ovarian serous adenocarcinoma,^44^ our study suggested that NAAG was a potential therapeutic target for high-risk BLIS tumors as well.

Several limitations of our study should be considered. First, as metabolomic detection technology is developing, the selection of internal standards, the choosing of mass spectrometers and the setting of cutoff values during peak identification might be optimized in the future. We have utilized several methods, such as calculating MS/MS matching scores,^45^ conducting quantitative detection for crucial metabolites, to make our conclusions more reliable. In addition, the therapeutic targets revealed in our study were based on the analysis of multiomic data and functional experiments in preclinical models. Although the functional validation of the identified targets in PDO and PDX models mimicked the effect on patients to some extent, the use of these drugs in patients still needs further investigation.

In conclusion, using this large TNBC metabolomic dataset, we described the metabolomic landscape and heterogeneity of TNBCs. Furthermore, by combining genomic, transcriptomic and metabolomic data, we identified several subtype-specific metabolomic therapeutic targets for TNBCs, which might expand the frontiers of our previous genomic-based precision medicine of TNBC.

## Materials and methods

### Patient cohort

Patients diagnosed with malignant breast cancer and who were willing to participate in the present study were retrospectively selected. Detailed sample selection was described in our previous study.^4^ In this study, we collected samples in our TNBC cohort with adequate tissues for polar metabolite and lipid detection. In all, 330 TNBC samples with 149 matched normal tissues were available for further detection. All tissue samples included in the study were obtained after approval of the research by the FUSCC Ethics Committee, and each patient provided written informed consent.

### Sample preparation and metabolomic detecting

Details of sample pretreatment, polar metabolome and lipidome detection, data analysis, targeted metabolite detection and stable isotope tracing analysis are included in the Supplementary information, Data S1.

### Categorization of polar metabolite and lipid

We referred to the KEGG database to categorize polar metabolites based on their KEGG metabolic pathways, resulting in eight categories: amino acids, carbohydrates, lipids, nucleotides, peptides, vitamins and cofactors, xenobiotics and others. For lipid data, we referred to the LIPID MAPS Structure Database (LMSD) to determine the categories and main classes. Five (fatty acyls [FA], glycerolipids [GL], glycerophospholipids [GP], sphingolipids [SP], sterol lipids [ST]) of the eight classical lipid categories were detected in our study.

### DA score

DA score captures the tendency for a pathway to have increased levels of metabolites, relative to a control group.^24^ The score is calculated by first applying a non-parametric DA test (in this study, Benjamini–Hochberg corrected Mann–Whitney *U* tests) to all metabolites in a pathway. Then, after determining which metabolites are significantly increased/decreased in abundance, the DA score is defined as:

$${{{{{{{\mathrm{DA}}}}}}}} = \frac{{{{{{{{{\mathrm{No}}}}}}}}.\;{{{{{{{\mathrm{of}}}}}}}}\;{{{{{{{\mathrm{metabolites}}}}}}}}\;{{{{{{{\mathrm{increased}}}}}}}} - {{{{{{{\mathrm{No}}}}}}}}.\;{{{{{{{\mathrm{of}}}}}}}}\;{{{{{{{\mathrm{metabolites}}}}}}}}\;{{{{{{{\mathrm{decreased}}}}}}}}}}{{{{{{{{{\mathrm{No}}}}}}}}.\;{{{{{{{\mathrm{of}}}}}}}}\;{{{{{{{\mathrm{measured}}}}}}}}\;{{{{{{{\mathrm{metabolites}}}}}}}}\;{{{{{{{\mathrm{in}}}}}}}}\;{{{{{{{\mathrm{pathway}}}}}}}}}}$$

Thus, the DA score varies from −1 to 1. A score of −1 indicates that all metabolites in a pathway decreased in abundance, while a score of 1 indicates that all metabolites increased.

The DA score can also be divided into two parts, i.e., the upregulated and the downregulated DA scores. The definitions are as follows:

$${{{{{{{\mathrm{Upregulated}}}}}}}}\;{{{{{{{\mathrm{DA}}}}}}}} = \frac{{{{{{{{{\mathrm{No}}}}}}}}.\;{{{{{{{\mathrm{of}}}}}}}}\;{{{{{{{\mathrm{metabolites}}}}}}}}\;{{{{{{{\mathrm{increased}}}}}}}}}}{{{{{{{{{\mathrm{No}}}}}}}}.\;{{{{{{{\mathrm{of}}}}}}}}\;{{{{{{{\mathrm{measured}}}}}}}}\;{{{{{{{\mathrm{metabolites}}}}}}}}\;{{{{{{{\mathrm{in}}}}}}}}\;{{{{{{{\mathrm{pathway}}}}}}}}}}$$

$${{{{{{{\mathrm{Downregulated}}}}}}}}\;{{{{{{{\mathrm{DA}}}}}}}} = \frac{{{{{{{{{\mathrm{No}}}}}}}}.\;{{{{{{{\mathrm{of}}}}}}}}\;{{{{{{{\mathrm{metabolites}}}}}}}}\;{{{{{{{\mathrm{decreased}}}}}}}}}}{{{{{{{{{\mathrm{No}}}}}}}}.\;{{{{{{{\mathrm{of}}}}}}}}\;{{{{{{{\mathrm{measured}}}}}}}}\;{{{{{{{\mathrm{metabolites}}}}}}}}\;{{{{{{{\mathrm{in}}}}}}}}\;{{{{{{{\mathrm{pathway}}}}}}}}}}$$

### Metabolomic clustering

Both metabolite and lipid data were pre-processed before clustering based on SNF.^28^ Only metabolites/lipids with significant tumor-normal differences (false discovery rate (FDR) < 0.01; | log_2_ fold change| > 1) were retained. These metabolites/lipids were further filtered with standard deviation (SD). Metabolites with the top 200 SDs and lipids with the top 400 SDs were kept for downstream SNF clustering. Three was identified as the optimal number of clusters using function “estimateNumberOfClustersGivenGraph” in R package “SNFtools” (both Eigen-gap best and rotation cost best). The clustering results were further checked using a similarity matrix and visualized using a network.

### Metabolomic and transcriptomic matching at the individual reaction level

Recon3D (an updated and expanded human metabolic network reconstruction) was used to identify pairs of genes/metabolites.^29^ The Recon3D dataset offered a large quantity of biochemical reactions with information on substrates, products, related catalyzing genes and the reversibility of the reactions. We first focused on the polar metabolites and the matching metabolic genes. The genes, whose products catalyze the reactions, were mapped to the corresponding substrates and products. Some uncommon reactions were also included. Detailed matching information was provided in Supplementary information, Table S7. A similar matching method was also mentioned in a published metabolomic study.^24^ As lipids usually consist of different lengths of fatty acyl chains and different glycosyl and phosphate groups, the biochemical reactions of lipid metabolism are complex. Several isozymes catalyzing similar biochemical reactions exist, but with distinct specificity to different lengths of fatty acyl chains or different glycosyl and phosphate groups. Therefore, it is difficult to precisely match lipids with their specific and paired metabolic genes.

### Analysis of the associations between somatic mutations and polar metabolomics and lipidomics

We applied a linear regression model to evaluate the associations between somatic mutations and polar metabolomics and lipidomics of TNBC according to a previous study.^26^ Metabolomic subtypes, tumor size, number of positive lymph nodes, age and BMI were adjusted to diminish the cofounding effect. Only one metabolite was included in the regression model at one time, in which the covariates were metabolomic subtyping information, tumor size, number of positive lymph nodes, age and BMI. The detailed formula of the model was as follows:

$$\begin{array}{l}{{{{{{{\mathrm{logit}}}}}}}}\left( {\pi \left( {{{{{{{{\mathrm{Y}}}}}}}} = 1} \right)} \right) = \beta 0 + \beta 1 \times \left( {{{{{{{{\mathrm{one}}}}}}}}\;{{{{{{{\mathrm{metabolite}}}}}}}}} \right) + \beta 2 \times \left( {{{{{{{{\mathrm{metabolomic}}}}}}}}\;{{{{{{{\mathrm{subtype}}}}}}}}} \right) + \\ \beta 3 \times \left( {{{{{{{{\mathrm{tumor}}}}}}}}\;{{{{{{{\mathrm{size}}}}}}}}} \right) + \beta 4 \times \left( {{{{{{{{\mathrm{number}}}}}}}}\;{{{{{{{\mathrm{of}}}}}}}}\;{{{{{{{\mathrm{positive}}}}}}}}\;{{{{{{{\mathrm{lymph}}}}}}}}\;{{{{{{{\mathrm{nodes}}}}}}}}} \right) + \beta 5 \times \left( {{{{{{{{\mathrm{age}}}}}}}}} \right) + \\ \beta 6 \times \left( {{{{{{{{\mathrm{BMI}}}}}}}}} \right)\end{array}$$

logit(π(Y = 1)): the mutation status of one gene; 0 or 1.

Known cancer-related genes^30,31^ that were mutated in at least 6% of the cases of at least one metabolic subtype were included. Lipidomic data were summarized based on the 47 subclasses, and the mean log_2_ abundance of lipids belonging to the same subclass was calculated as the abundance of this lipid subclass. Each mutation variable was converted to binary indicator (1/0) in our analysis. The calculated T-statistics and associated *P*-values were reported to evaluate the associations. The mutation features were scored by associations with each metabolite and can be compared based on statistical significances.

### Analysis of the associations between SCNAs and polar metabolomics and lipidomics

The correlation between SCNAs and metabolite abundance was calculated with a linear regression model. Metabolomic subtypes, tumor size, number of positive lymph nodes, age and BMI were adjusted to diminish the cofounding effect. Lipidomic data were also summarized as described in the mutation-metabolite correlation section. T-statistics and associated *P* values were calculated for SCNA peak and metabolite pairs. SCNA peak-metabolite pairs included 97 GISTIC peaks and metabolites. GISTIC peaks were obtained from the file “all_lesions.conf_95.txt” resulting from GISTIC2.0 as previously described.^4^

### Analysis of the association between mRNA expression of cancer-related genes and polar metabolomics and lipidomics

The correlation between the mRNA expression of cancer-related genes and metabolite abundance was calculated with a linear regression model. Details were described in the sections for mutations and SCNAs. The list of cancer-related genes was derived from the network of cancer gene datasets.^30^ Oncogenes that were confirmed in breast cancer were included. Then, genes with significant differences in mRNA expression between TNBC samples and normal tissues (|log_2_FC| > 1, FDR < 0.01) were selected for further analysis. In all, 27 cancer-related genes were included in the analysis.

### Simplification of the metabolomic subtyping system for BLIS tumors with machine-learning methods

The bootstrap method was used to construct the discovery and test cohort. The metabolomic subtyping model was developed in the discovery cohort via two different methods: 1) multivariate linear regression (R package “glm”), 2) support vector machine (R package “e1071”). The least absolute shrinkage and selection operator (Lasso) method was used to select the most useful predictive features from the training cohort.^46^ Tuning parameter (*λ*) selection in the LASSO model used 5-fold cross-validation. The ability to predict TNBC subtypes was assessed by the area under the curve (AUC) of the ROC curve in the test cohort via the R package “pROC”.^47^ Comparisons of AUCs were determined using the R package “pROC”. The contribution of each predictor (metabolite) in the LASSO model is defined by:

$${{{{{{{\mathrm{Contribution}}}}}}}} = \frac{{|{{{{{{{\mathrm{coefficient}}}}}}}}_i|}}{{{\sum} {|{{{{{{{\mathrm{coefficient}}}}}}}}_i|} }}$$

i: the metabolite included in the linear model

### Human TNBC cell lines

All human TNBC cell lines were purchased from American Type Culture Collection. Each cell line identity was verified by short tandem repeat profiling. Cells were grown in complete growth medium as previously described.^48^ Only cells that were thawed within 6 months were used for the current study. To ensure the maintenance of phenotypes, cell morphology and doubling times were also regularly recorded.

### Identification of the transcriptomic subtypes, metabolic-gene-based subtypes and metabolomic subtypes for TNBC cell lines

Transcriptomic subtyping and metabolic-gene-based subtyping were based on the RNA-seq data of our cohort. Metabolic-gene-based metabolic pathway scores were first calculated with the ssGSEA method (R package “GSVA”). Then, the TNBC cell lines were included in the t-sne analysis (R package “Rtsne”) for transcriptomic subtyping and metabolic-gene-based subtyping. Metabolomic subtyping was based on the metabolomic data of the CCLE dataset (https://portals.broadinstitute.org/ccle).^26^ The subtyping method was similar to that of metabolic-gene-based subtyping.

### shRNAs and transfection

HEK293T cells were transfected with shRNA vector and packaging plasmid mix using Neofect DNA transfection reagents (Tengyi Biotech, #TF201201). The supernatant containing virus was collected 48 h after transfection with a 0.45-μm filter. Targeted cells were infected with shRNA lentivirus with 8 µg/mL polybrene (Sigma, #H9268) and then selected with 2 µg/mL of puromycin (Sangon Biotech, #A610593-0025) for one week. The shRNA primers are as follows:

sh#1 F:CCGGCTGAAGTTCTGGAGTTCCCAACTCGAGTTGGGAACTCCAGAACTTCAGTTTTTG;

sh#1 R:AATTCAAAAACTGAAGTTCTGGAGTTCCCAACTCGAGTTGGGAACTCCAGAACTTCAG

sh#2 F:CCGGGAAGAGATAGAGCATGACATACTCGAGTATGTCATGCTCTATCTCTTCTTTTTG;

sh#2 R:AATTCAAAAAGAAGAGATAGAGCATGACATACTCGAGTATGTCATGCTCTATCTCTTC

The efficiency of silencing was assessed by immunoblotting. For immunoblotting analysis, cells were lysed in modified RIPA buffer (50 mM Tris-HCl, pH 7.4, 1% Nonidet P-40, 0.25% sodium deoxycholate, 150 mM NaCl, and 1 mM EDTA). Protein concentrations were determined using BCA protein assay reagent (Yeasen, #20201ES90). Cell extracts were subjected to SDS-PAGE, transferred to PVDF membranes (Millipore, #IPVH00010), and incubated with the indicated primary antibodies (RIMKLB: Proteintech, 26111-1-AP; vinculin:Sigma, V9131).

### Small interfering RNA (siRNA)

For *SPHK1* siRNA transduction, LM2-4175 and MDA-MB-453 cells were transfected with *SPHK1* siRNA and negative control siRNA using Lipofectamine™ 3000 Reagent (Lipo3000, Invitrogen, California, USA) 48 h before the drug treatment. The details of the siRNA are as follows:

**Table:** 

siRNA		5′→3′ sequence
si*SPHK1*#1	sense strand	GCA GGC AUA UCG AGU AUG ATT
	antisense strand	UCA UAC UCC AUA UGC CUG CTT
si*SPHK1*#2	sense strand	CCA UGA ACC UGC UGU CUC UTT
	antisense strand	AGA GAC AGC AGG UUC AUG GTT
siSPHK1#NC	sense strand	UUC UCC GAA CGU GUC ACG UTT
	antisense strand	ACG UGA CAC GUU CGG AGA ATT

The siRNA and negative control siRNA constructs were synthesized by Guangzhou Ruibo Biotechnology Co., Ltd. (Guangzhou, China). The knockdown efficiency was verified through quantitative PCR (qPCR) by using specific primers (F: 5′–GCTCTGGTGGTCATGTCTGG–3′, R: 5′–CACAGCAATAGCGTGCAGT–3′).

### Cell proliferation assay

Cell proliferation assays were performed as previously described.^49^ The DNA content of the cells was determined using a Fluorescent DNA Quantitation kit (Bio-Rad Laboratories, Hercules, CA). For each analysis, three replicate wells were used, and at least three independent experiments were performed.

### Cell migration and invasion assays

Cell migration was measured in a Boyden chamber using Transwell filters obtained from Corning (Cambridge, MA). Cells (3–10 × 10^5^) in 0.2 mL serum-free medium were placed in the upper chamber, and the lower chamber was loaded with 0.8 mL medium containing 10% FBS. Cells that migrated to the lower surface of filters were stained with Wright Giemsa solution, and five fields of each well were counted after 24 h of incubation at 37 °C with 5% CO_2_. Three wells were examined for each condition and cell type, and the experiments were repeated in triplicate. Cell invasion assays were performed using a Chemicon cell invasion kit (Chemicon International, Temecula, CA) in accordance with the manufacturer’s protocol. Cells (3–10 × 10^5^/mL) were seeded onto 24-well cell culture chamber using inserts with an 8 lM pore size polycarbonate membrane over a thin layer of extracellular matrix. Following incubation of the plates for 48 h at 37 °C, cells that had invaded through the ECM layer and migrated to the lower surface of the membrane were stained and counted under the microscope in at least 10 different fields and photographed.

### Fuel dependency assay of cell lines

The mitochondrial fuel dependency of each cell line was measured using a Seahorse XF Mito Fuel Flex Test Kit (Seahorse Biosciences, #103260-100) following the manufacturer’s protocols. Briefly, cells were plated in Seahorse XF96 well plates at proper intensity (~3 × 10^4^ cells/well) and incubated overnight. On the next day, the culture medium was replaced with XF24 DMEM containing 10 mM glucose, 2 mM glutamine and 1 mM pyruvate. The mitochondrial oxygen consumption rate (OCR) was measured at basal levels as well as with drugs inhibiting the utilization of the substrate. The final concentrations of the various inhibitors used were as follows: mitochondrial pyruvate carrier inhibitor (UK5099), 2 mM; Glutaminease I inhibitor (BPTES), 3 mM; carnitine palmitoyltransferases 1 A inhibitor (Etomoxir), 4 mM. The dependency for use of one of the three substrates was assessed by OCR changes with the addition of one inhibitor and the OCR changes after the addition of all three inhibitors. The dependency of each fuel was calculated using the following formulas:

$${{{{{{{\mathrm{Dependency}}}}}}}}\;\left( {{{{{{{\mathrm{\% }}}}}}}} \right) = \frac{{OCR_{the\;first\;injection} - OCR_{the\;second\;injection}}}{{OCR_{the\;first\;injection} - OCR_{the\;last\;injection}}} \times 100$$

### Isotopic labeling

For serine tracing, 1 × 10^7^ cells were cultured for 24 h in serine/glycine-free medium (Teknova). Then, 42 mg/L [U-^13^C3,^15^N]-serine (CNLM-474-H, Cambridge Isotope Laboratory) was added to the medium and cells were cultured for another 24 h. Then, cells were washed and collected for analysis. Details of the isotopic labeling measurement are included in Supplementary information, Data S1.

### Compounds

PF-543 (S7177), Opaganib (S7174), Siponimod (S7179) and JTE 013 (S7182) were purchased from Selleck. NCT-503 (HY-101966), FTY-720 (HY-12005) and SK-IN-1 (HY-101805) were purchased from MedChemExpress. NAAG (A5930) was purchased from Sigma.

### Identification of the transcriptomic subtypes of TNBC for PDO and PDX models

The TNBC transcriptomic subtype of each PDO or PDX was determined by our previously defined IHC methods.^33^ In brief, AR, CD8, FOXC1 and DCLK1 were utilized as markers for TNBC subtyping. This IHC method has been widely used in the clinical setting in our center. Therefore, we can directly acquire the subtype for the TNBC patient from the pathological report.

### Organoid preparation and culture

We developed a biobank for organoid storage as previously described.^50,51^ Fresh breast cancer tissues were placed in cold DMEM/F12 (Gibco) with primocin (InvivoGen) and transported to the lab in an ice box for tumor cell isolation and culture. Tissues were washed in cold PBS 2–3 times and then minced into small fragments (1 mm^3^ or less) using sterile scalpels. Tissues were digested with collagenase and hyaluronidase in digestion buffer (DMEM/F12 with 5% BSA, insulin and hydrocortisone) for 12 h at 37 °C. Dissociated tissues were spun down at 350× *g* for 5 min and resuspended in 10 mL of Tris-NH4Cl buffer, incubated for 3 min to remove red blood cells and passed through a 100 μm cell strainer (Corning). Dissociated cell clusters were centrifuged for 5 min at 350× *g* and resuspended in digestion buffer and spun down again. Cell clusters were resuspended in BME type-2 buffer (Trevigen, 3533-010-02) and plated as a 300 μL drop within a 12 mm, 0.4 μm inner Transwell chamber (Corning). The drop was solidified by a 30-min incubation at 37 °C and 5% CO_2_ with 1 mL of breast cancer organoid medium (Advanced DMEM/F12 supplemented with R-spondin-1 [500 ng/mL, Peprotech], Noggin [100 ng/mL, Peprotech], Neuregulin [5 nM, Peprotech], Estradiol [5 nM, Sigma], HEPES [1 mM, Gibco], GlutaMAX [1×, Gibco], Nicotinamide [5 mM, Sigma], N-Acetylcysteine [1.25 mM, Sigma], B-27 [1×, Gibco]. A83-01 [0.5 mM, Tocris], Primocin [1×, InvivoGen], SB-202190 [500 nM, Selleck], Y27632 [5 uM, Selleck], FGF10 [20 ng/mL, Peprotech], FGF7 [5 ng/mL, Peprotech] and EGF [5 ng/mL, Peprotech]). For passaging, 5 mL harvesting solution (Trevigen, 3700-100-01) was used to digest the BME and incubated on ice for 1 h. Subsequently, organoids were centrifuged at 350× *g* for 5 min, washed in digestion buffer and spun down. 3 mL TrypLE Express (InvitroGen) was added, and organoids were incubated at room temperature for 3 min, followed by mechanical dissociation to small cell clusters by pipetting. Organoids were passaged at a 1:2–3 dilution every 2–3 weeks. All PDOs included in the study were obtained after approval of the research by the FUSCC Ethics Committee, and each patient provided written informed consent.

### Drug response test of TNBC organoids

For organoid drug treatment, organoids in good condition were harvested and digested into single cells. Organoids were diluted to 40 organoids/µL in breast cancer organoid medium containing 10% BME.^51^ 25 µL organoid suspension was added to cell-repellent black surface, clear bottom 384-well plates (Greiner 781976-SIN) and cultured for another 5–6 days before drug treatments. For glycine/serine depletion culture, organoids were cultured for 2 weeks before testing for viability. Organoid cell viability was evaluated by a CellTiter-Glo 3D cell viability assay (Promega, G9683) according to the manufacturer’s instructions.

### Drug response test of TNBC mini patient-derived xenograft (mini-PDX) models

To rapidly test drug efficacy in vivo, we established mini-PDX models according to previous papers.^52,53^ Tumor cells derived from PDO models were harvested and digested into single cells. Cells were then filled into OncoVee® capsules (LIDE Biotech, Shanghai, China). Each capsule contained ~2000 cells. Capsules were implanted subcutaneously via a small skin incision with 3 capsules per mouse (5-week-old female nu/nu mouse). Mice bearing MiniPDX capsules were treated with appropriate control or drugs (PF-543 and FTY-720). PF-543 and FTY-720 were administered via tail vein injection, as single administrations (Daily [qd] × 1) for 7 continuous days at doses of 5 mg/kg or 1 mg/kg, respectively. All these drugs were prepared by being dissolved in DMSO, PEG300 and Tween-80 solutions. Vehicle controls were isometric 0.5% HPMC and 0.2% Tween-80 solution and the vehicle treatment was performed the same way as drug treatment. After all capsules were removed from mice, tumor cell proliferation in each capsule was measured using the CellTiter Glo Luminescent Cell Viability Assay kit (G7571, Promega, Madison, WI, US). Tumor cell growth inhibition rate was calculated using the published formula.^53^

### In vivo mouse studies

Five- to six-week-old female NOD/SCID mice were obtained from Shanghai Jihui Laboratory Animal Care Co. Ltd. A total of 1 × 10^6^ LM2-4175 breast cancer cells with or without *RIMKLB* knockdown were injected subcutaneously into the mammary fat pad region of mice. Tumor size was measured twice or three times weekly using a caliper. Tumor volume in mm^3^ was calculated using the formula: tumor volume = 0.5 × L × W^2^, where L is the longest dimension and W is the perpendicular dimension. After 14 days, each group was divided into two groups: supplement with or without NAAG. The mice bearing tumors were injected with NAAG daily, via intraperitoneal injection at a dose of 50 mg/kg.^43^ Mice were sacrificed at day 31. All animal experiments were performed according to protocols approved by the Research Ethical Committee of Fudan University Shanghai Cancer Center. The protocols of all animal experiments were reviewed and approved by Institutional Animal Care and Use Committee (FUSCC-IACUC-2021381).

### Statistical analysis

Two-tailed Student’s *t-*test, Wilcoxon’s test and Kruskal–Wallis test were utilized to compare continuous variables and ordered categorical variables. Prior to the comparisons, the normality of the distributions was tested with the Shapiro–Wilk test before comparison. Pearson’s chi-square test or Fisher’s exact test was employed for the comparison of unordered categorical variables. A permutation test was conducted to compare gene mutation frequencies among clusters. Correlation matrices were created with Pearson’s or Spearman’s correlation. RFS was defined as the time from diagnosis to first recurrence, a diagnosis of contralateral breast cancer or death of any cause. Patients without events were censored from the time point of the last follow-up. Survival analysis was performed using the Kaplan-Meier method, and the survival of the clusters was compared using the log rank test. All the tests were two-sided, and *P* < 0.05 indicates significance, unless otherwise stated. The FDR correction was utilized in multiple tests to decrease false positive rates. All of the analyses were performed with R software (version 3.4.2, http://www.R-project.org).

## Supplementary information

Supplementary Data S1

Fig. S1

Fig. S2

Fig. S3

Fig. S4

Fig. S5

Fig. S6

Fig. S7

Fig. S8

Fig. S9

Fig. S10

Supplementary Tables

## Data Availability

The polar metabolomic and lipidomic data of our cohort are provided in Supplementary information, Tables S1–S4. The accession number for raw LC-MS data, microarray data and sequence data reported in this paper is NODE: OEP000155. All data can be viewed in The National Omics Data Encyclopedia (NODE) (http://www.biosino.org/node) by pasting the accession (OEP000155) into the text search box or through the URL: http://www.biosino.org/node/project/detail/OEP000155.
