# Supplementary material for: Comprehensive metabolomics expands precision medicine for triple-negative breast cancer
Source: Cell Res. 2022 Feb 1;32(5):477–90. doi: 10.1038/s41422-022-00614-0 (PMC9061756; doi:10.1038/s41422-022-00614-0)
Supplement: Supplementary file 1 — Supplementary Data S1 [file 41422_2022_614_MOESM1_ESM.pdf]

Supplementary information, Data S1

Metabolomic detection methods

1. Polar metabolomics detection

1.1 Sample quenching and extraction

For polar metabolites extraction, 25 mg tissue from each sample was mixed with 1000 μL extract solution (acetonitrile: methanol: water = 2: 2: 1) containing 2 μg/mL L-2-chlorophenylalanine as an internal standard. We pre-weighed the sample to ensure that similar weight of each sample was used for the subsequent process. After 30 s of vortexing, the samples were homogenized at 40 Hz for 4 min, sonicated for 10 min in ice-water and repeated for 3 cycles. Then the samples were incubated at -40 °C for 1 h and centrifuged at 10000 rpm for 15 min at 4 °C. 800 μL of supernatant was transferred to a fresh tube and dried in a vacuum concentrator at 37 °C. The dried samples were reconstituted in 200 μL of 50% acetonitrile by sonication on ice for 10 min and the supernatants were collected after centrifugation at 13000 rpm for 15 min at 4 °C and 75 μL of supernatant was transferred to a fresh glass vial for LC-MS analysis [1]. The quality control (QC) sample was prepared by mixing an equal aliquot of the supernatants from all of the samples.

1.2 Chromatography separation

For metabolomics data collection, a 1290 Infinity series UHPLC System (Agilent Technologies, USA) equipped with a UPLC BEH Amide column (2.1 * 100 mm, 1.7 μm, Waters) was used. Mobile phase A consisted of 25 mmol/L ammonium acetate and 25 mmol/L ammonia hydroxide in water (pH = 9.75).

The mobile phase B consisted of acetonitrile. The elution gradient was set as follows: 0~0.5 min, 95% B; 0.5~7.0 min, 95%~65% B; 7.0~8.0 min, 65%~40% B; 8.0~9.0 min, 40% B; 9.0~9.1 min, 40%~95% B; 9.1~12.0 min, 95% B. The column temperature was 25°C. The auto-sampler temperature was 4 °C, and the injection volumes in both positive and negative ion mode were 2 μL.

1.3 Mass spectrometry

Tumor, normal, quality control (QC) and blank samples were performed for acquiring MS spectra using 6550 Q-TOF mass spectrometer (Agilent Technologies). ESI source conditions were set as following: Gas Temperature as 250 °C, Gas Flow as 16 L/min, Sheath Gas Temperature as 350 °C, Sheath Gas Flow as 12 L/min, Nebulizer as 20 psi, Fragmentor as 175 V, Capillary Voltage as 3000 V. A Triple TOF 6600 mass spectrometer (Sciex) was used to acquire MS/MS spectra on an information-dependent acquisition (IDA) mode. QC samples were used for MS/MS data acquisition. In this mode, the acquisition software (Analyst TF 1.7, Sciex) continuously evaluates the full scan survey MS data as it collects and triggers the acquisition of MS/MS spectra depending on preselected criteria. In each cycle, 12 precursor ions with intensity greater than 100 were chosen for MS/MS analysis at collision energy (CE) of 30 eV. The cycle time was 0.56 s. ESI source conditions were set as follows: Gas 1 as 60 psi, Gas 2 as 60 psi, Curtain Gas as 35 psi, Source Temperature as 600 °C, Declustering potential as 60 V, Ion Spray Voltage Floating (ISVF) as 5000 V or -4000 V in positive or negative ion modes, respectively. To ensure that the metabolic features from 6550 Q-TOF and Triple TOF 6600 are the same, we matched the features with the following standards: m/z difference less than 25 p.p.m and retention time difference less than 30 s.

Our study only included MS and MS/MS matched and MS/MS confirmed features for further analysis [2-5].

1.4 Data quality control

Internal standards (ISs), QC and blank samples were used to evaluate the instrument variability during the whole process of detecting. In terms of ISs, instrument variability was determined by calculating the relative standard deviation (RSD) for the ISs that were added to each sample prior to injection in the mass spectrometers. RSD value of ISs were shown in the table below.

Internal standard	Median RSD_{QC}
Polar metabolites detection	5.51%

For QC samples, as described before, after the pretreatment of samples, an equal aliquot (10 μL) of the supernatants from all of the samples was extracted and mixed together. During the whole detecting process, the QC replicate samples were injected every eight samples; these QC samples were treated independently throughout the process as if they were client study samples. Then, the distribution of QC samples in principal component analysis were illustrated to evaluate the process variability (**Supplementary information, Figure S1**).

1.5 Data processing, metabolite identification and data analysis

MS raw data files were converted to the mzXML format by ProteoWizard, and processed by R package XCMS (version 3.2). The process includes peak deconvolution, alignment and integration. The processing results generated a data matrix that consisted of the retention time (RT), mass-to-charge ratio (m/z) values, and peak abundance. To make the metabolomics data reproducible, the relative standard derivation (RSD) of the peaks in the QC samples larger

than 30% were filtered out. The remaining peaks were annotated by comparison to retention time and mass to charge ratio (m/z) indices in the library by using the R package CAMERA [6]. After that, we obtained a data matrix consisting of the retention time, m/z and peak intensities. The data matrix was further processed by removing the peaks with missing values (intensity = 0) in more than 50% of the samples. For the remaining peaks, the missing values were replaced with 50% of the lowest observed value of all detected samples [7,8]. The area of each peak was then normalized by the total ion current for the polar metabolomics [9]. To remove the unwanted analytical variations occurring intra- and inter batches, each metabolite peak in all subject samples was normalized using the LOESS method based on QC samples. In brief, a LOESS regression model was built based on the intensity drift of each metabolite in the QC samples and was used to predict and correct intensities of the same metabolite in subject samples [9]. In all, 10992 MS features were included for further annotation.

As described in the mass spectrometry section, the MS and MS/MS spectra were matched according to their accurate masses (±25 ppm), and RT values (±30 s). Only matched MS and MS/MS spectra were included for the further annotation.

Then the MS/MS spectra were searched in an in-house database (BiotreeDB) for polar metabolite annotation based on accurate mass (m/z, ± 25 ppm), retention time and spectral patterns [10-13]. The MS/MS spectra matching score was calculated using dot-product algorithm, which take the fragments and intensities into consideration [14]. Metabolites with MS/MS matching score higher than 0.3 were included in our study. In all, 594 MS/MS features were annotated

and included in our study. The median m/z errors of these metabolites were 9.86 ppm, 50.8% of the m/z errors were less than 10 ppm, and 93.4% of the m/z errors were less than 20 ppm.

In summary, our study took several steps to ensure the accuracy of the metabolite identification. only the peaks that were matched between the two mass spectrometers, with MS/MS name and with MS/MS matching score higher than 0.3 were included for further analysis [2-5].

2. Lipidomics detection

2.1 Sample quenching and extraction

For lipid extraction, 20 mg tissue of each sample was used. Lipids were extracted by adding 200 μL water and 480 μL extract solution (MTBE: MeOH = 5: 1) sequentially. 0.625 ug/ml 15:0-18:1-d7-PE, 18:1-d7 Lyso PC, 15:0-18:1-d7-PE and 18:1-d7 Lyso PC were added as internal standards. We pre-weighed the sample to ensure that similar weight of each sample was used for the subsequent process. After 30 s vortex, the samples were homogenized at 40 Hz for 4 min and sonicated for 10 min in ice-water bath. The homogenization and sonication cycle was repeated for 3 times. Then the samples were incubated at -40 °C for 1 h and centrifuged at 10000 rpm for 15 min at 4 °C. 300 μL of supernatant was transferred to a fresh tube and dried in a vacuum concentrator at 37 °C. Then, the dried samples were reconstituted in 100 μL of 50% methanol in dichloromethane by sonication on ice for 10 min. The constitution was then centrifuged at 13000 rpm for 15 min at 4 °C, and 75 μL of supernatant was transferred to a fresh glass vial for LC-MS analysis [15,16]. The quality control (QC) sample was prepared by mixing an equal aliquot of the

supernatants from all of the samples.

2.2 Chromatography separation

For lipidomics data collection, an ExionLC Infinity series UHPLC System (Sciex) with a Kinetex C18 column (2.1 * 100 mm, 1.7 μm, Phenomen) coupled with a triple TOF 5600 mass spectrometer (Sciex) was used. Briefly, samples were analyzed in both positive and negative ion modes. The mobile phase A consisted of 60% acetonitrile in water, and 10 mmol/L ammonium formate. The mobile phase B consisted of 10% acetonitrile and 90% isopropanol with 0.5 mmol/L ammonium formate. The elution gradient as set as follows: 0~12.0 min, 40%~100% B; 12.0~13.5 min, 100% B; 13.5~13.7 min, 100%~40% B; 13.7~18.0 min, 40% B. The column temperature was 45 °C. The auto-sampler temperature was 4 °C, and the injection volume was 2 μL in both positive and negative ion mode.

2.3 Mass spectrometry

The Triple TOF 5600 mass spectrometer was used for its ability to acquire MS and MS/MS spectra on an information-dependent basis (IDA) during the LC-MS and LC-MS/MS experiments. In this mode, the acquisition software (Analyst TF 1.7, Sciex) continuously evaluates the full scan survey MS data as it collects and triggers the acquisition of MS/MS spectra depending on preselected criteria. In each cycle, 12 precursor ions with intensity above 100 were chosen for MS/MS analysis at collision energy (CE) of 45 eV (12 MS/MS events with accumulation time of 50 msec each). ESI source conditions were set as following: Gas 1 as 60 psi, Gas 2 as 60 psi, Curtain Gas as 30 psi, Source Temperature as 600 °C, Declustering potential as 100 V, Ion Spray Voltage Floating (ISVF) as 5000 V or -3800 V in positive or negative modes,

respectively.

2.4 Data quality control

Internal standards (ISs), QC and blank samples were used to evaluate the instrument variability during the whole process of detecting. In terms of ISs, instrument variability was determined by calculating the relative standard deviation (RSD) for the ISs that were added to each sample prior to injection in the mass spectrometers. RSD value of ISs were shown in the table below.

**Internal standard**	**Median RSD_{QC}**
Lipid detecting	14.27%

For QC samples, as described before, after the pretreatment of samples, an equal aliquot (10 μL) of the supernatants from all of the samples was extracted and mixed together. During the whole detecting process, the QC replicate samples were injected every eight samples; these QC samples were treated independently throughout the process as if they were client study samples. Then, the distribution of QC samples in principal component analysis were illustrated to evaluate the process variability (**Supplementary information, Figure S1**).

2.5 Data processing, metabolite identification and data analysis

Unlike the analysis for polar metabolite, lipidomics has another procedure for analysis. The raw data files (.wiff format) were converted to files in mzXML format using the ‘msconvert’ program from ProteoWizard (version 3.0.19282). Then, the mzxML files were loaded into LipidAnalyzer for data processing. Peak detection was first applied to the MS data. The CentWave algorithm in XCMS was used for peak detection. To make the lipidomic data reproducible, the relative standard derivation (RSD) of the peaks in the QC samples larger than

30% were filtered out. The remaining peaks were annotated by comparison to retention time and mass to charge ratio (m/z) indices in the library by using the R package CAMERA [17]. After that, we obtained a data matrix consisting of the retention time, m/z and peak intensities. The data matrix was further processed by removing the peaks with missing values (intensity = 0) in more than 50% of the samples. For the remaining peaks, the missing values were replaced with 50% of the lowest observed value of all detected samples [7,8]. The area of each peak was then normalized by the total ion current for the lipidomic datasets [9]. To remove the unwanted analytical variations occurring intra- and inter batches, each metabolite peak in all subject samples was normalized using the LOESS method based on QC samples. In briefly, a LOESS regression model was built based on the intensity drift of each metabolite in the QC samples and was used to predict and correct intensities of the same metabolite in subject samples [9]. In all, 14348 MS features were included for further annotation.

With the MS/MS spectrum, lipid identification was achieved through a spectral match using the LipidBlast database with the reverse matching method [18]. The MS/MS tolerance were set at 25 ppm [11-13]. The MS/MS spectra matching score was also calculated using dot-product algorithm, which take the fragments and intensities into consideration [14]. Metabolites with MS/MS matching score higher than 0.3 were included in our study. In all, 1944 features were annotated and included in our study. The median m/z errors of these metabolites were 6.89 ppm, 60.9% of the m/z errors were less than 10 ppm, and 86.4% of the m/z errors were less than 20 ppm.

In summary, only the peaks with MS/MS name and with MS/MS matching score higher than 0.3 were included in our study for further analysis.

3. Targeted detecting and absolute quantitation of metabolites

3.1 Targeted detection of sphingosine, sphinganine, sphingosine-1-phosphate

An aliquot of each individual sample was precisely weighed and transferred to an Eppendorf tube. A 1000 μL aliquot of methanol: acetonitrile: water (2:2:1 volume ratio) was added to the remaining sample solution with vortex. The samples were vortexed for 30 s, homogenized at 45 Hz for 4 min, and sonicated for 10 min in ice-water bath with one big and two small steel balls. The homogenate and sonicate circle was repeated for 3 times, followed by subsiding at -40 °C for 1 h and centrifuging at 12000 rpm and 4 °C for 15 min. An 800 μL aliquot of the supernatant was used for vacuum drying. A 100 μL aliquot of 50% acetonitrile was added for resolution. After vortex mixing for 30 s, the resolution was sonicated for 10 min in ice-water bath. The samples were centrifuged at 12000 rpm and 4 °C for 15 min. The 80 μL supernatant was used for UHPLC-MS-MS analysis. The UHPLC separation was carried out using an EXIONLC System (Sciex), equipped with a Waters ACQUITY UPLC® HSS T3 (100 × 2.1 mm, 1.8 μm, Waters). Mobile phase A consisted of 5 mmol/L ammonium formate and 0.1% formic acid in water, and mobile phase B consisted of 5 mmol/L ammonium formate and 0.1% formic acid in 95% acetonitrile. The column temperature was set at 35 °C. The auto-sampler temperature was set at 4 °C and the injection volume was 2 μL. A SCIEX 6500 QTRAP+ triple quadrupole mass spectrometer (Sciex), equipped with an IonDrive Turbo V electrospray ionization (ESI) interface, was applied for assay development. Typical ion source parameters were: Curtain Gas = 40 psi, IonSpray Voltage = 4500 V, temperature = 475 °C, Ion Source Gas 1 = 30 psi,

Ion Source Gas 2 = 30 psi. The MRM parameters for each of the targeted analytes were optimized using flow injection analysis, by injecting the standard solutions of the individual analytes, into the API source of the mass spectrometer. Several of the most sensitive transitions were used in the MRM scan mode to optimize the collision energy for each Q1/Q3 pair. Among the optimized MRM transitions per analyte, the Q1/Q3 pairs that showed the highest sensitivity and selectivity were selected as ‘quantifier’ for quantitative monitoring. The additional transitions acted as ‘qualifier’ for the purpose of verifying the identity of the target analytes. Quantitative analysis were carried out in multiple reaction monitoring (MRM) on transitions m/z 300.3/282.2, 300.3/252.3, 300.3/264.4 for sphingosine, m/z 302.3/284.2, 302.3/254.3, and 302.3/266.2 for sphinganine and m/z 380.2/362.3, 380.2/247.3, and 380.2/264.4 for sphingosine-1-phosphate. We verified the corresponding results of the three MRM transitions and selected m/z 300.3/252.3 for sphingosine, m/z 302.3/254.3 for sphinganine and m/z 380.2/264.4 for sphingosine-1-phosphate for quantitation, respectively. SCIEX Analyst Work Station Software (Version 1.6.3) and Sciex MultiQuant™ 3.0.3 were employed for MRM data acquisition and processing.

3.2 Targeted detection of NAAG

For each sample, 1000 μL extract solution (methanol: acetonitrile: water = 2: 2: 1) containing internal standard was added. After 30 s vortex, the samples were homogenized at 35 Hz for 4 min and sonicated for 10 min in ice-water bath. The homogenization and sonication cycle was repeated for 3 times. Then the samples were incubated at -40 °C for 1 h and centrifuged at 12000 rpm for 15 min at 4 °C. 100 μL of supernatant was transferred to a fresh glass vial for

LC-MS/MS analysis. The UHPLC separation was carried out using an EXIONLC System (Sciex), equipped with a Waters ACQUITY UPLC® Amide (100 × 2.1 mm, 1.7 μm, Waters). The mobile phase A consisted of 25 mmol/L ammonium acetate and 25 mmol/L ammonium hydroxide in water, and the mobile phase B consisted of acetonitrile. The column temperature was set at 30°C. The auto-sampler temperature was set at 4°C and the injection volume was 1 μL. A SCIEX 6500 QTRAP+ triple quadrupole mass spectrometer (Sciex), equipped with an IonDrive Turbo V electrospray ionization (ESI) interface, was applied for assay development. Typical ion source parameters were: Curtain Gas = 40 psi, IonSpray Voltage = -4500 V, temperature = 475°C, Ion Source Gas 1 = 30 psi, Ion Source Gas 2 = 30 psi. The MRM parameters for each of the targeted analytes were optimized using flow injection analysis, by injecting the standard solutions of the individual analytes, into the API source of the mass spectrometer. Several most sensitive transitions were used in the MRM scan mode to optimize the collision energy for each Q1/Q3 pair. Among the optimized MRM transitions per analyte, the Q1/Q3 pairs that showed the highest sensitivity and selectivity were selected as 'quantifier' for quantitative monitoring. The additional transitions acted as 'qualifier' for the purpose of verifying the identity of the target analytes. Quantitative analysis were carried out in multiple reaction monitoring (MRM) on transitions m/z 303.1/285.1, 303.1/128/0 and 303.1/95.9 for NAAG. We chose m/z 305/128.0 for quantitation of NAAG. SCIEX Analyst Work Station Software (Version 1.6.3) was employed for MRM data acquisition and processing.

3.3 The preparation of the calibration curves

Stock solutions were individually prepared by dissolving or diluting each

standard substance to give a final concentration of 10 mmol/L. An aliquot of each of the stock solutions was transferred to a 10 mL flask to form a mixed working standard solution. A series of calibration standard solutions were then prepared by stepwise dilution of this mixed standard solution (containing internal standard in identical concentrations with the samples). Calibration solutions were subjected to UPLC-MRM-MS/MS analysis using the methods described above. The least squares method was used for the regression fitting. 1/x weighting was applied in the curve fitting since it provided highest accuracy and correlation coefficient (R2). The level was excluded from the calibration if the accuracy of calibration was not within 80%–120%.

3.4 Quantification of metabolites

Quantification in TNBC samples was as follows: the final concentration (C_F, nmol/L) equals the calculated concentration (C_C, nmol/L) multiplied by the dilution factor (Dil). The metabolite concentration (C_M, nmol/Kg) equals the final concentration (C_F, nmol/L) multiplied by the final volume (V_F, μL), and divided by the weight (M_S, mg) of the sample.

$$c_M[\text{nmol} \cdot kg^{-1}] = \frac{c_F[\text{nmol} \cdot L^{-1}] \cdot V_F[\mu L]}{Ms[mg]}$$

Quantification in TNBC cell lines was as follows: the final concentration (C_F, nmol/L) equals the calculated concentration (C_C, nmol/L) multiplied by the dilution factor (Dil). The metabolite concentration (C_M, nmol/cell) equals the final concentration (C_F, nmol/L) multiplied by the final volume (V_F, μL), and divided by the number (Ns, number) of the cells.

$$c_M[\text{nmol} \cdot cell^{-1}] = \frac{c_F[\text{nmol} \cdot L^{-1}] \cdot V_F[\mu L] \times 10^{-6}}{Ns[cell\ \ number]}$$

4. LC-MS analysis of serine and stable isotope tracing

Stable-isotope labeling of cells were previously described. The pretreatment of the collected cells were as follows: after the addition of 200 μL of water, the samples were vortexed for 30 s. The samples were precooled in dry ice, repeated freeze-thaw three times in liquid nitrogen. 50 μL of the sample was mixed with 150 μL water, and then 480 μL extract solution (MTBE: methanol = 5:1, containing internal standard) was added. After 60 s vortex, the samples were sonicated for 10 min in ice-water bath. Then the samples were centrifuged at 3000 rpm for 15 min at 4°C. 250 μL of supernatant was transferred to a fresh tube. The rest of the sample was added with 250 μL of MTBE, followed with vortex, sonication and centrifugation, and another 250 μL of supernatant was taken out. This step was repeated twice. And the supernatants were combined and dried under a gentle stream of nitrogen. Then, the dried samples were reconstituted in 200 μL of resuspension buffer (DCM:MeOH:H2O= 60:30:4.5) by sonication on ice for 10 min. The constitution was then centrifuged at 12000 rpm for 15 min at 4 °C, and 30 μL of supernatant was transferred to a fresh glass vial for LC-MS analysis.

The UHPLC separation was carried out using a SCIEX ExionLC series UHPLC System. The mobile phase A consisted of 40% water, 60% acetonitrile, and 10 mmol/L ammonium formate. The mobile phase B consisted of 10% acetonitrile and 90% isopropanol, and 10 mmol/L ammonium formate. The column temperature was 45 °C. The auto-sampler temperature was 4 °C, and the injection volume was 2 μL. Sciex QTrap 6500+ mass spectrometer was applied for assay development. Typical ion source parameters were: IonSpray Voltage: +5500/-4500 V, Curtain Gas: 40 psi, Temperature: 350 °C, Ion Source

Gas 1:50 psi, Ion Source Gas 2: 50 psi, DP: ±80 V. Downstream products of ceramide pathway of *de novo* synthesis and degradation were analyzed with the proportion of isotope labeling.

5. TNBC metabolomic data transformation

All metabolomic data with further analysis in this study is multiplied by 10^9 and then $\log_2$-transformed.

Reference

1. Yang, C., *et al.* Effect of vitamin D3 on immunity and antioxidant capacity of pearl oyster Pinctada fucata martensii after transplantation: Insights from LC-MS-based metabolomics analysis. *Fish Shellfish Immunol* **94**, 271-279 (2019).
2. Mei, X., *et al.* RIPK1 regulates starvation resistance by modulating aspartate catabolism. *Nat Commun* **12**, 6144 (2021).
3. Liang, L., *et al.* Metabolic Dynamics and Prediction of Gestational Age and Time to Delivery in Pregnant Women. *Cell* **181**, 1680-1692 e1615 (2020).
4. Lu, J., *et al.* Comprehensive metabolomics identified lipid peroxidation as a prominent feature in human plasma of patients with coronary heart diseases. *Redox Biol* **12**, 899-907 (2017).
5. Jia, H., *et al.* Predicting the pathological response to neoadjuvant chemoradiation using untargeted metabolomics in locally advanced rectal cancer. *Radiother Oncol* **128**, 548-556 (2018).
6. Kuhl, C., Tautenhahn, R., Bottcher, C., Larson, T.R. & Neumann, S. CAMERA: an integrated strategy for compound spectra extraction and annotation of liquid chromatography/mass spectrometry data sets. *Anal Chem* **84**, 283-289 (2012).
7. Wei, R., *et al.* Missing Value Imputation Approach for Mass Spectrometry-based Metabolomics Data. *Sci Rep* **8**, 663 (2018).
8. Tiedt, S., *et al.* Circulating Metabolites Differentiate Acute Ischemic Stroke from Stroke Mimics. *Ann Neurol* **88**, 736-746 (2020).
9. Dunn, W.B., *et al.* Procedures for large-scale metabolic profiling of serum and plasma using gas chromatography and liquid chromatography coupled to mass spectrometry. *Nat Protoc* **6**, 1060-1083 (2011).
10. Shen, X., *et al.* Metabolic reaction network-based recursive metabolite annotation for untargeted metabolomics. *Nat Commun* **10**, 1516 (2019).
11. Zhou, Z., *et al.* Ion mobility collision cross-section atlas for known and unknown metabolite annotation in untargeted metabolomics. *Nat Commun* **11**, 4334 (2020).
12. Li, T., *et al.* Ion mobility-based sterolomics reveals spatially and temporally distinctive sterol lipids in the mouse brain. *Nat Commun* **12**, 4343 (2021).
13. Lv, J., *et al.* A serum metabolomics analysis reveals a panel of screening metabolic biomarkers for esophageal squamous cell carcinoma. *Clin Transl Med* **11**, e419 (2021).
14. Stein, S.E. & Scott, D.R. Optimization and testing of mass spectral library search algorithms for compound identification. *J Am Soc Mass Spectrom* **5**, 859-866 (1994).
15. Matyash, V., Liebisch, G., Kurzchalia, T.V., Shevchenko, A. & Schwudke, D. Lipid extraction by methyl-tert-butyl ether for high-throughput lipidomics. *J Lipid Res* **49**, 1137-1146 (2008).
16. Tu, J., Yin, Y., Xu, M., Wang, R. & Zhu, Z.J. Absolute quantitative lipidomics reveals lipidome-wide alterations in aging brain. *Metabolomics* **14**, 5 (2017).
17. XueKe, G., *et al.* Lipidomics and RNA-Seq Study of Lipid Regulation in Aphis gossypii parasitized by Lysiphlebia japonica. *Sci Rep* **7**, 1364 (2017).
18. Kind, T., *et al.* LipidBlast in silico tandem mass spectrometry database for lipid identification. *Nat Methods* **10**, 755-758 (2013).
