# Supplementary material for: Comprehensive metabolomics expands precision medicine for triple-negative breast cancer
Source: Cell Res. 2022 Feb 1;32(5):477–90. doi: 10.1038/s41422-022-00614-0 (PMC9061756; doi:10.1038/s41422-022-00614-0)
Supplement: Supplementary file 2 — Fig. S1 [file 41422_2022_614_MOESM2_ESM.pdf]

Fig. S1

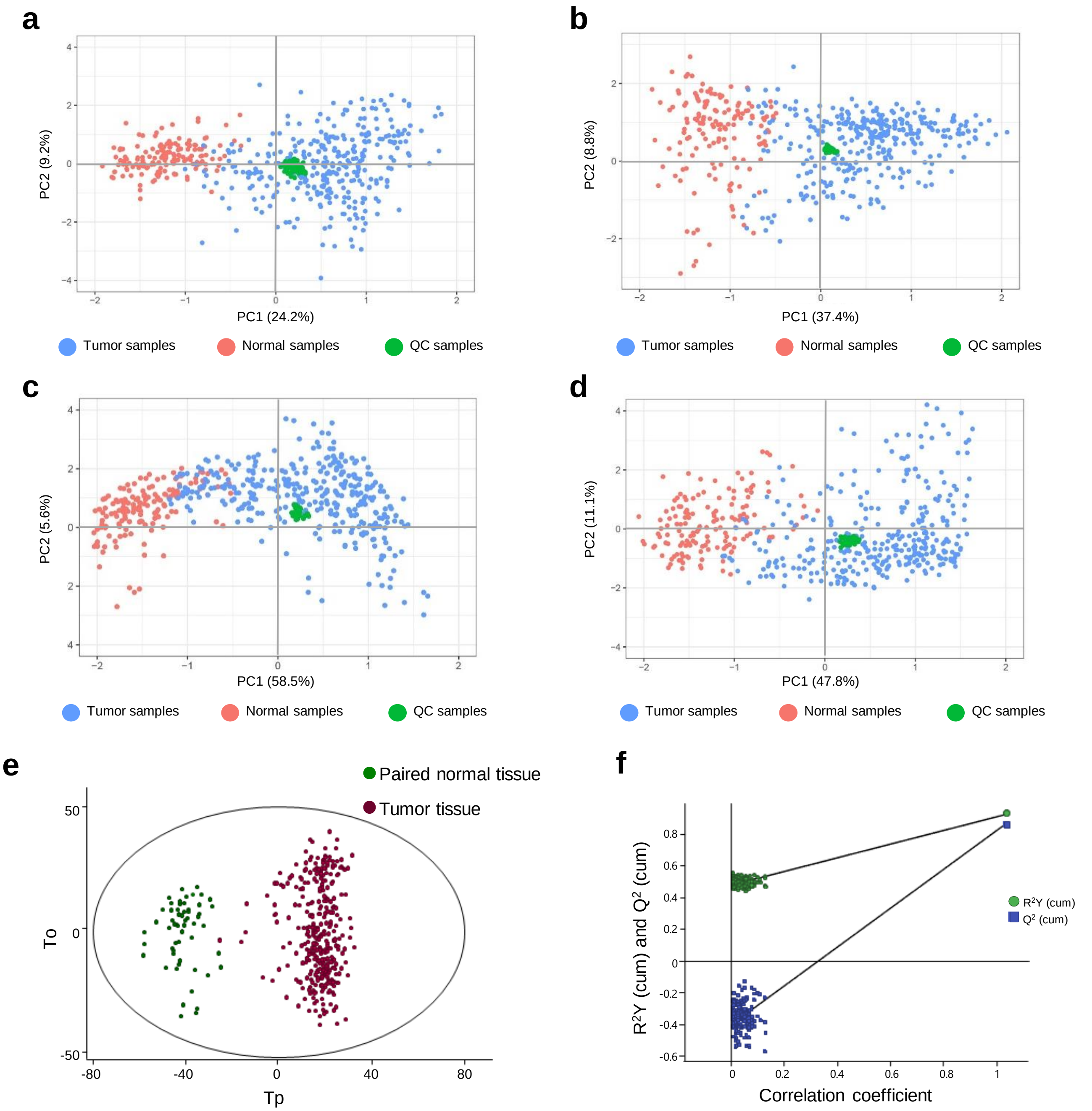

Fig. S1. Quality control of the polar metabolomic and lipidomic data

a, b The distribution of quality control (QC) samples in principal component analysis (PCA) plots of polar metabolomic data in positive- (a) and negative- (b) ion modes. **c, d** The distribution of QC samples in PCA plots of lipidomic data in positive- (c) and negative- (d) ion modes. MS/MS-verified metabolites were included for PCA plots and the Pareto method was utilized for scaling. Tumor and paired normal samples were annotated in the PCA plots. **e**, **f** The OPLS-DA analysis of metabolomic data. The distribution of the tumor and the normal samples in the plot of OPLS-DA analysis (**e**) and the robustness of the OPLS-DA model (**f**) were illustrated.
