# Supplementary material for: Comprehensive metabolomics expands precision medicine for triple-negative breast cancer
Source: Cell Res. 2022 Feb 1;32(5):477–90. doi: 10.1038/s41422-022-00614-0 (PMC9061756; doi:10.1038/s41422-022-00614-0)
Supplement: Supplementary file 3 — Fig. S2 [file 41422_2022_614_MOESM3_ESM.pdf]

Fig. S2

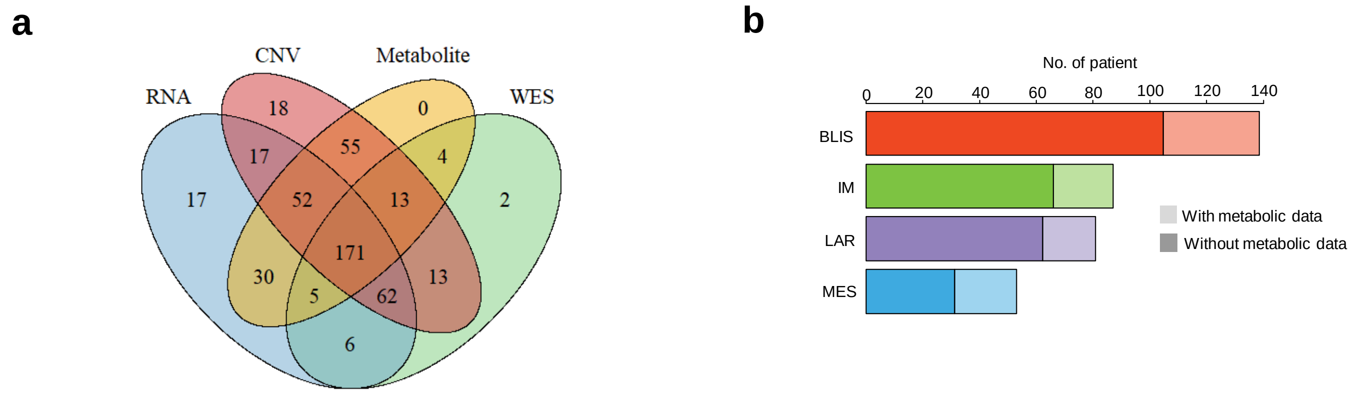


**Fig. S2. Sample information of the cohort**

**a** Venn diagram of the studied samples with the multiomic data. **b** Number of patients having metabolomic data in each TNBC transcriptomic subtype.
