# Supplementary material for: Comprehensive metabolomics expands precision medicine for triple-negative breast cancer
Source: Cell Res. 2022 Feb 1;32(5):477–90. doi: 10.1038/s41422-022-00614-0 (PMC9061756; doi:10.1038/s41422-022-00614-0)
Supplement: Supplementary file 4 — Fig. S3 [file 41422_2022_614_MOESM4_ESM.pdf]

Fig. S3

a

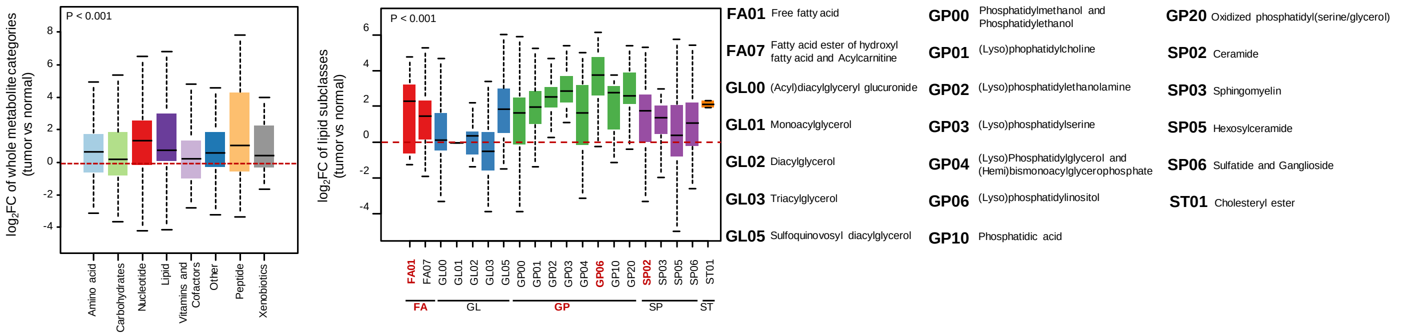

P < 0.001
log2FC of whole metabolite categories (tumor vs normal)
Amino acid
Carbohydrates
Nucleotide
Lipid
Vitamins and Cofactors
Other
Peptide
Xenobiotics
P < 0.001
log2FC of lipid subclasses (tumor vs normal)
FA01 FA07 GL00 GL01 GL02 GL03 GL05 GP00 GP01 GP02 GP03 GP04 GP06 GP10 GP20 SP02 SP03 SP05 SP06 ST01
FA
GL
GP
SP
ST
FA01 Free fatty acid
FA07 Fatty acid ester of hydroxyl fatty acid and Acylcarnitine
GL00 (Acyl)diacylglyceryl glucuronide
GL01 Monoacylglycerol
GL02 Diacylglycerol
GL03 Triacylglycerol
GL05 Sulfoquinovosyl diacylglycerol
GP00 Phosphatidylmethanol and Phosphatidylethanol
GP01 (Lyso)phophatidylcholine
GP02 (Lyso)phosphatidylethanolamine
GP03 (Lyso)phosphatidylserine
GP04 (Lyso)Phosphatidylglycerol and (Hemi)bismonoacylglycerophosphate
GP06 (Lyso)phosphatidylinositol
GP10 Phosphatidic acid
GP20 Oxidized phosphatidyl(serine/glycerol)
SP02 Ceramide
SP03 Sphingomyelin
SP05 Hexosylceramide
SP06 Sulfatide and Ganglioside
ST01 Cholesteryl ester

b

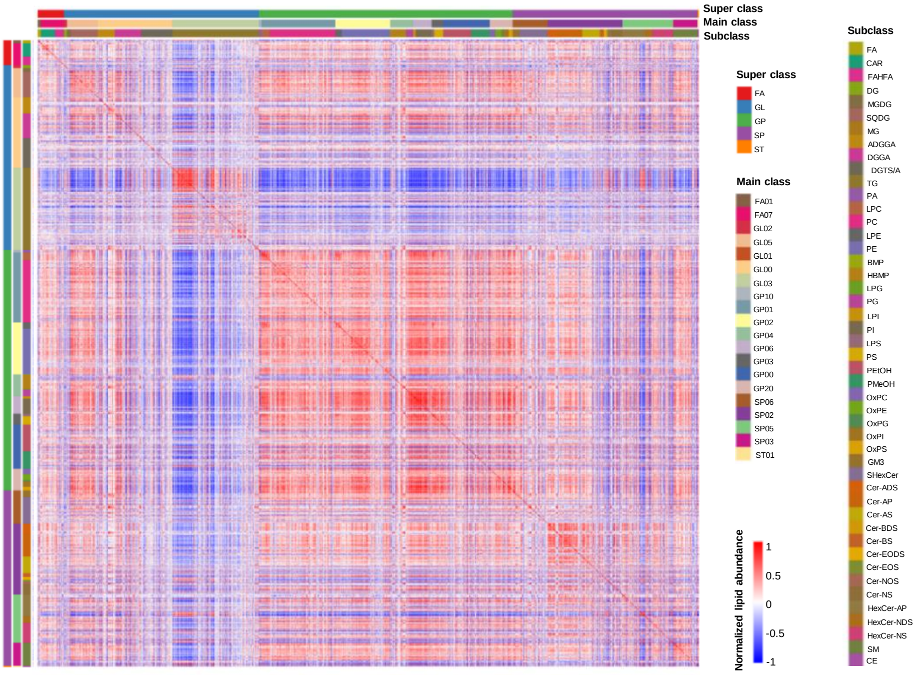

Super class
Main class
Subclass
Super class
FA
GL
GP
SP
ST
Main class
FA01 FA07 GL02 GL05 GL01 GL00 GL03 GP10 GP01 GP02 GP04 GP06 GP03 GP00 GP20 SP06 SP02 SP05 SP03 ST01
Normalized lipid abundance
1
0.5
0
-0.5
-1
Subclass
FA CAR FAHFA DG MGDG SQDG MG ADGGA DGGA DGTS/A TG PA LPC PC LPE PE BMP HBMP LPG PG LPI PI LPS PS PEtOH PMeOH OxPC OxPE OxPG OxPI OxPS GM3 SHexCer Cer-ADS Cer-AP Cer-AS Cer-BDS Cer-BS Cer-EODS Cer-EOS Cer-NOS Cer-NS HexCer-AP HexCer-NDS HexCer-NS SM CE

c

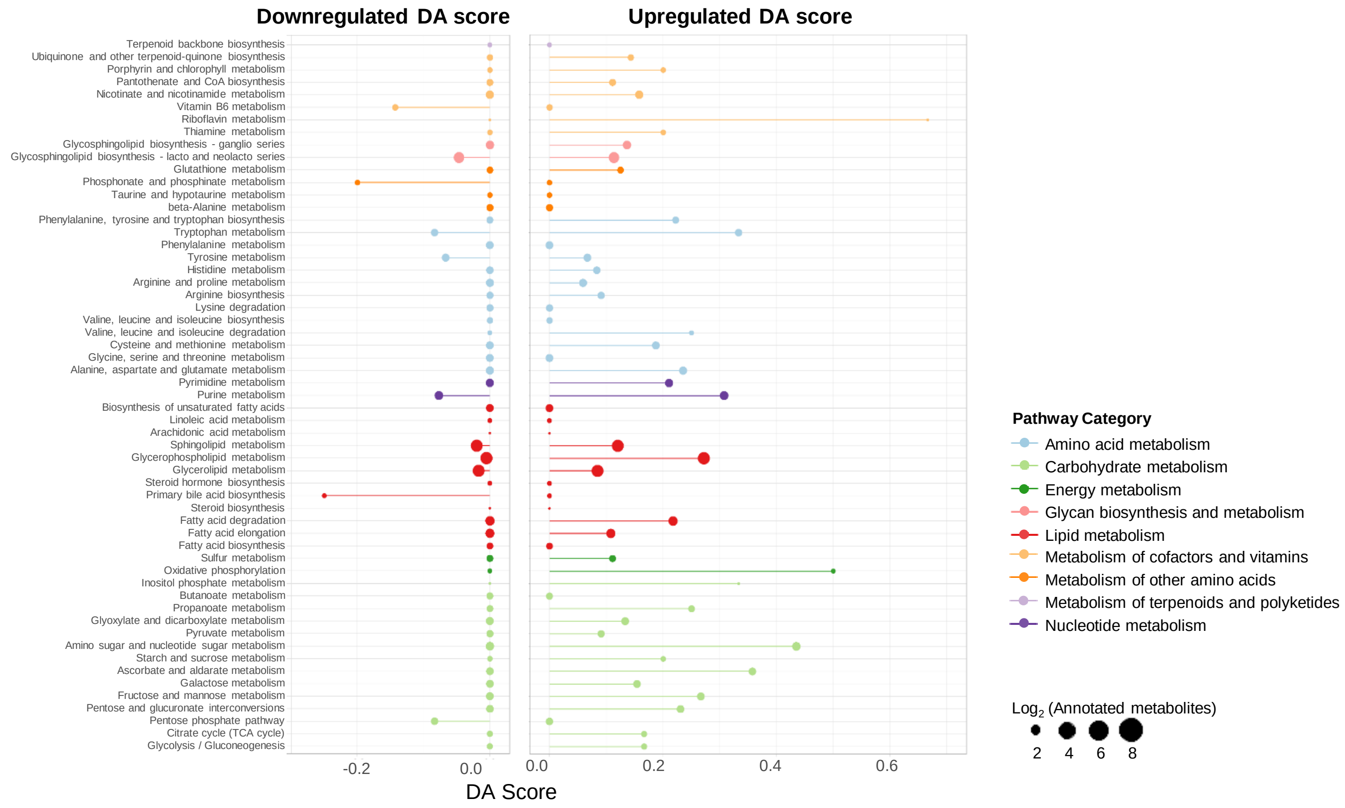

Downregulated DA score
Upregulated DA score
Terpenoid backbone biosynthesis
Ubiquinone and other terpenoid-quinone biosynthesis
Porphyrin and chlorophyll metabolism
Pantothenate and CoA biosynthesis
Nicotinate and nicotinamide metabolism
Vitamin B6 metabolism
Riboflavin metabolism
Thiamine metabolism
Glycosphingolipid biosynthesis - ganglio series
Glycosphingolipid biosynthesis - lacto and neolacto series
Glutathione metabolism
Phosphonate and phosphinate metabolism
Taurine and hypotaurine metabolism
beta-Alanine metabolism
Phenylalanine, tyrosine and tryptophan biosynthesis
Tryptophan metabolism
Phenylalanine metabolism
Tyrosine metabolism
Histidine metabolism
Arginine and proline metabolism
Arginine biosynthesis
Lysine degradation
Valine, leucine and isoleucine biosynthesis
Valine, leucine and isoleucine degradation
Cysteine and methionine metabolism
Glycine, serine and threonine metabolism
Alanine, aspartate and glutamate metabolism
Pyrimidine metabolism
Purine metabolism
Biosynthesis of unsaturated fatty acids
Linoleic acid metabolism
Arachidonic acid metabolism
Sphingolipid metabolism
Glycerophospholipid metabolism
Glycerolipid metabolism
Steroid hormone biosynthesis
Primary bile acid biosynthesis
Steroid biosynthesis
Fatty acid degradation
Fatty acid elongation
Fatty acid biosynthesis
Sulfur metabolism
Oxidative phosphorylation
Inositol phosphate metabolism
Butanoate metabolism
Propanoate metabolism
Glyoxylate and dicarboxylate metabolism
Pyruvate metabolism
Amino sugar and nucleotide sugar metabolism
Starch and sucrose metabolism
Ascorbate and aldarate metabolism
Galactose metabolism
Fructose and mannose metabolism
Pentose and glucuronate interconversions
Pentose phosphate pathway
Citrate cycle (TCA cycle)
Glycolysis / Gluconeogenesis
-0.2
0.0
0.0
0.2
0.4
0.6
DA Score
Pathway Category
Amino acid metabolism
Carbohydrate metabolism
Energy metabolism
Glycan biosynthesis and metabolism
Lipid metabolism
Metabolism of cofactors and vitamins
Metabolism of other amino acids
Metabolism of terpenoids and polyketides
Nucleotide metabolism
Log2 (Annotated metabolites)
2
4
6
8

Fig. S3. Supplementary information of the TNBC metabolomic landscape

a $\log_2$ fold changes of the abundances of polar metabolites and lipids between TNBC tumor and normal tissues were summarized in the boxplot based on categories. The $\log_2$ FC value of 0 (dashed red line) indicates the same level of metabolite abundance between the tumor and the normal. **b** Correlations of lipids based on their abundance. The categories of lipids were summarized and annotated both in rows and in columns. **c** DA scores were classified into two parts, i.e. the upregulated and the downregulated DA scores, to reflect the general upregulation and downregulation of metabolic pathways.
