# Supplementary material for: Comprehensive metabolomics expands precision medicine for triple-negative breast cancer
Source: Cell Res. 2022 Feb 1;32(5):477–90. doi: 10.1038/s41422-022-00614-0 (PMC9061756; doi:10.1038/s41422-022-00614-0)
Supplement: Supplementary file 5 — Fig. S4 [file 41422_2022_614_MOESM5_ESM.pdf]

Fig. S4

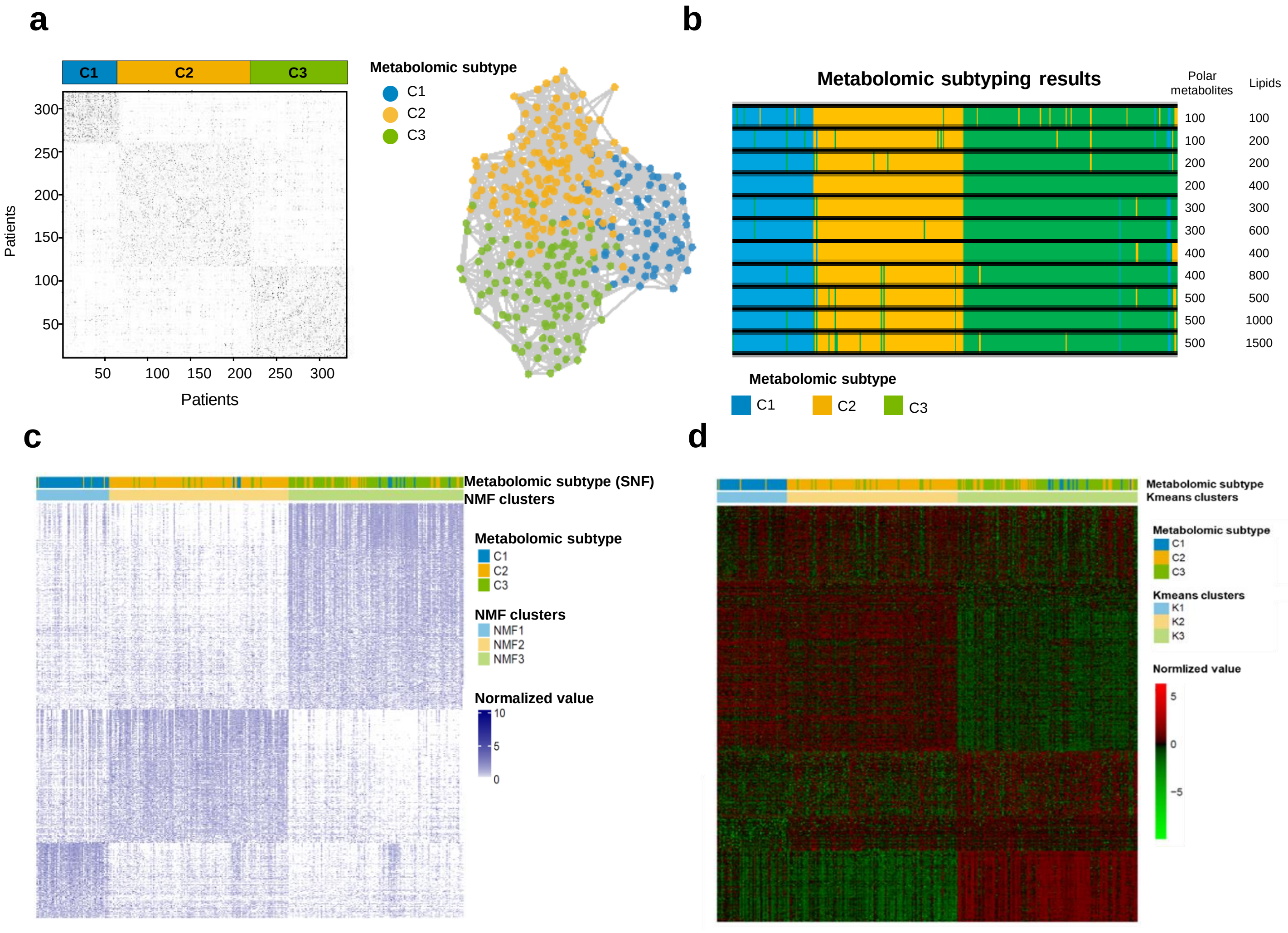

Fig. S4. Validation of TNBC metabolomic subtypes

a The discrimination of three metabolomic clusters based on SNF method. **b** SNF method-based metabolomic subtyping using different numbers of metabolites. Different cutoff values of top standard deviations (SDs) among tumor samples were utilized to select metabolites for subtyping. **c**, **d** TNBC metabolomic subtyping with non-negative matrix factorization (NMF) (**c**) and k-means (**d**) methods.
