# Supplementary material for: Comprehensive metabolomics expands precision medicine for triple-negative breast cancer
Source: Cell Res. 2022 Feb 1;32(5):477–90. doi: 10.1038/s41422-022-00614-0 (PMC9061756; doi:10.1038/s41422-022-00614-0)
Supplement: Supplementary file 6 — Fig. S5 [file 41422_2022_614_MOESM6_ESM.pdf]

Fig. S5

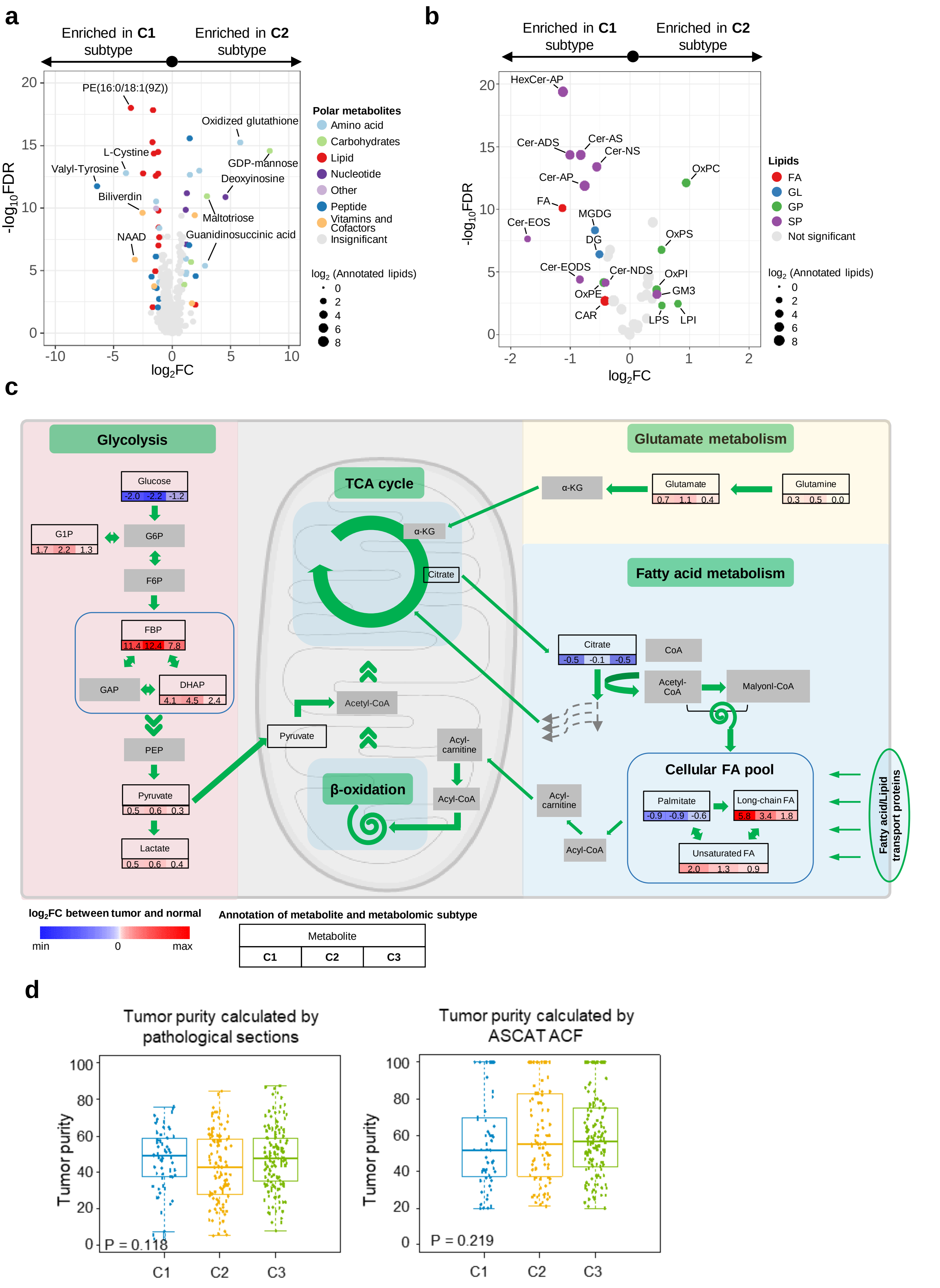

a
Enriched in C1 subtype
Enriched in C2 subtype
-log10FDR
log2FC
PE(16:0/18:1(9Z))
Oxidized glutathione
L-Cystine
Valyl-Tyrosine
GDP-mannose
Deoxyinosine
Biliverdin
Maltotriose
NAAD
Guanidinosuccinic acid
Polar metabolites
Amino acid
Carbohydrates
Lipid
Nucleotide
Other
Peptide
Vitamins and Cofactors
Insignificant
log2 (Annotated lipids)
b
Enriched in C1 subtype
Enriched in C2 subtype
-log10FDR
log2FC
HexCer-AP
Cer-ADS
Cer-AS
Cer-NS
Cer-AP
OxPC
FA
MGDG
Cer-EOS
DG
OxPS
Cer-EODS
Cer-NDS
OxPI
GM3
OxPE
CAR
LPS
LPI
Lipids
FA
GL
GP
SP
Not significant
log2 (Annotated lipids)
c
Glycolysis
Glucose
-2.0 -2.2 -1.2
G1P
1.7 2.2 1.3
G6P
F6P
FBP
11.4 12.4 7.8
GAP
DHAP
4.1 4.5 2.4
PEP
Pyruvate
0.5 0.6 0.3
Lactate
0.5 0.6 0.4
TCA cycle
α-KG
Citrate
Acetyl-CoA
Pyruvate
Acyl-carnitine
Acyl-CoA
β-oxidation
Glutamate metabolism
α-KG
Glutamate
0.7 1.1 0.4
Glutamine
0.3 0.5 0.0
Fatty acid metabolism
Citrate
-0.5 -0.1 -0.5
CoA
Acetyl-CoA
Malyonl-CoA
Cellular FA pool
Palmitate
-0.9 -0.9 -0.6
Long-chain FA
5.8 3.4 1.8
Unsaturated FA
2.0 1.3 0.9
Acyl-carnitine
Acyl-CoA
Fatty acid/Lipid transport proteins
log2FC between tumor and normal
min 0 max
Annotation of metabolite and metabolomic subtype
Metabolite
C1 C2 C3
d
Tumor purity calculated by pathological sections
Tumor purity
P = 0.118
C1 C2 C3
Tumor purity calculated by ASCAT ACF
Tumor purity
P = 0.219
C1 C2 C3

Fig. S5. Metabolomic features of the TNBC metabolomic subtypes

a, b $\log_2$ fold change of the abundances of polar metabolites (**a**) and lipid subclasses (**b**) between the C1 and C2 subtypes. The mean $\log_2$ fold change of the abundances of lipids belonging to one category was regarded as the $\log_2$ (fold change) of the lipid category. **c** Differences in energy metabolism among metabolomic subtypes. Metabolic changes in glycolysis, fatty acid metabolism and glutamate metabolism are illustrated. $\log_2$ fold change values of the indicated metabolites in tumor tissues as compared with normal tissues are shown. **d** Comparison of tumor purity among metabolomic subtypes. Tumor purity was evaluated using hematoxylin and eosin (H&E) stained slides (left) and aberrant cell fraction (ACF) calculated by ASCAT tool with copy number data (right).
