# Supplementary material for: Comprehensive metabolomics expands precision medicine for triple-negative breast cancer
Source: Cell Res. 2022 Feb 1;32(5):477–90. doi: 10.1038/s41422-022-00614-0 (PMC9061756; doi:10.1038/s41422-022-00614-0)
Supplement: Supplementary file 7 — Fig. S6 [file 41422_2022_614_MOESM7_ESM.pdf]

Fig. S6

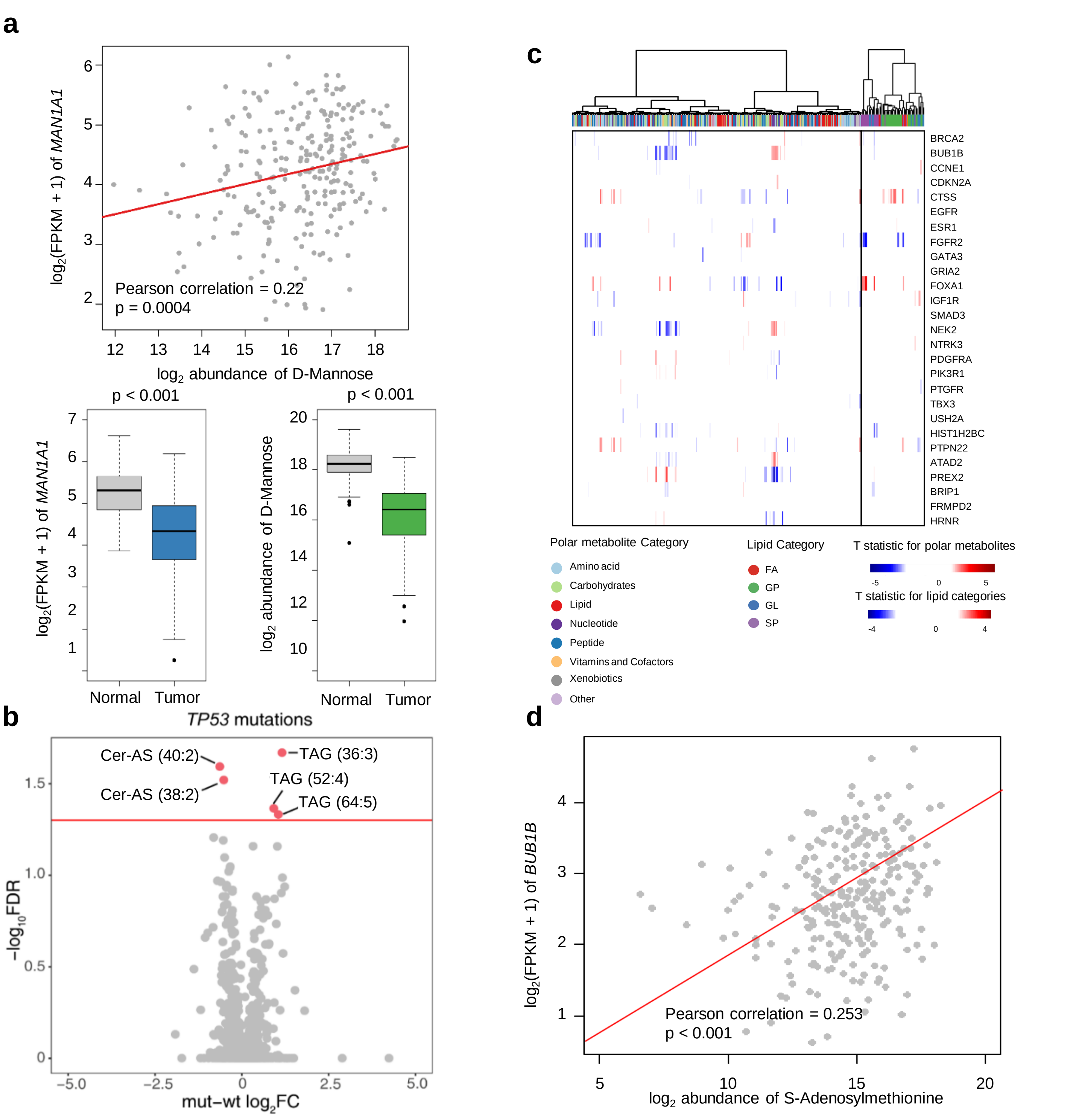

Fig. S6. The associations of polar metabolites and lipids with genomic features.

a Top panel: The correlation of D-mannose abundance with the mRNA expression of the catalyzing enzyme *MAN1A1*. Bottom panel: The comparison of mRNA expression of *MAN1A1* (left) and D-mannose abundance (right) between the tumor and the normal. **b** $\log_2$ fold change of the abundances of metabolites between *TP53* mutant and *TP53* wild type samples of TNBC. **c** Heatmap showing the correlations between the abundances of of metabolites (*y*-axis) and the mRNA expression of breast cancer-related genes (*x*-axis) for TNBC. T statistics were calculated by a linear regression model that adjusted the cofounding factors. **d** The correlation of S-Adenosylmethionine abundance with the mRNA expression of *BUB1B*.
