# Supplementary material for: Comprehensive metabolomics expands precision medicine for triple-negative breast cancer
Source: Cell Res. 2022 Feb 1;32(5):477–90. doi: 10.1038/s41422-022-00614-0 (PMC9061756; doi:10.1038/s41422-022-00614-0)
Supplement: Supplementary file 8 — Fig. S7 [file 41422_2022_614_MOESM8_ESM.pdf]

Fig. S7

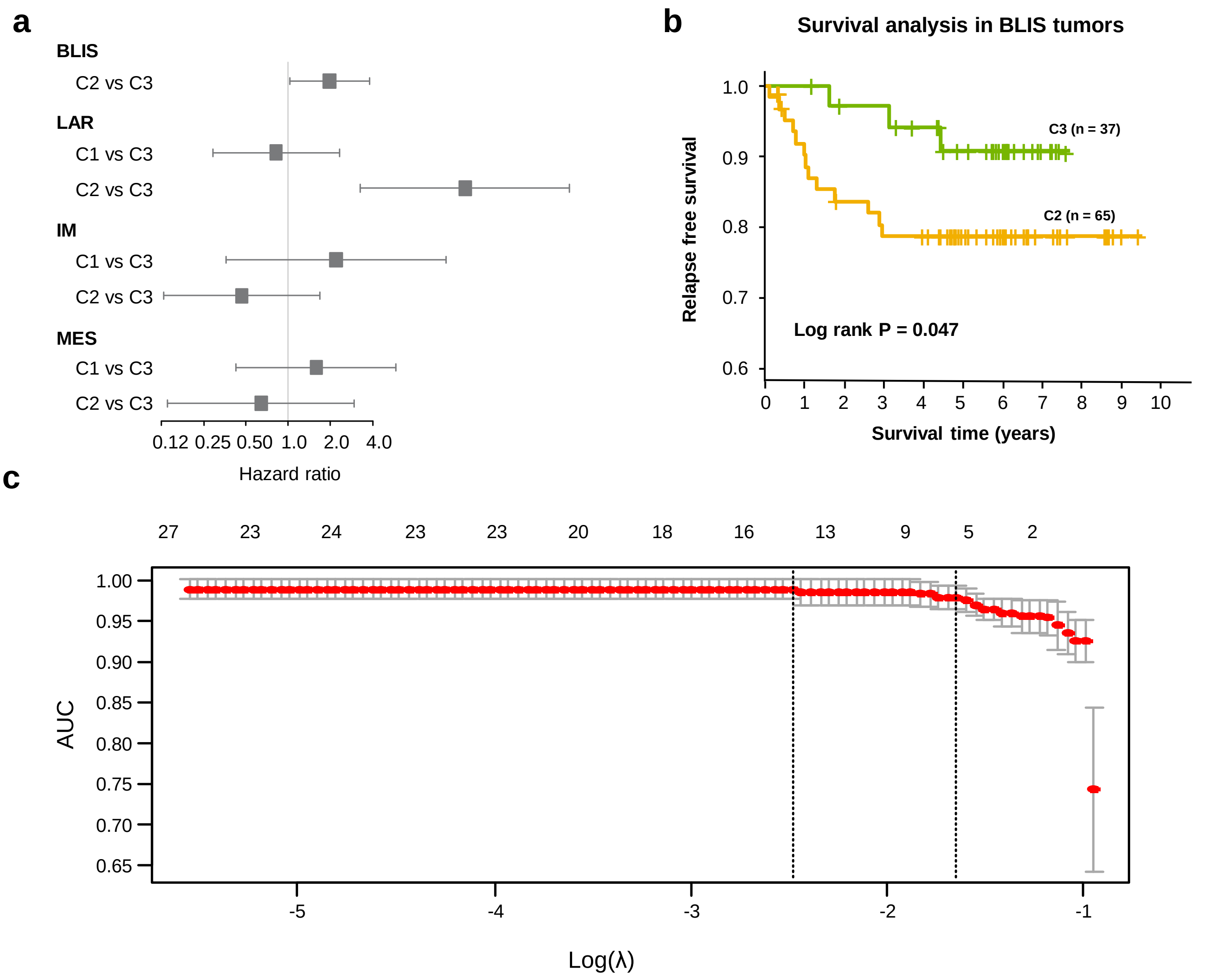

Fig. S7. The establishment of the machine learning model for metabolomic subtyping within BLIS tumors

a Prognostic value of metabolomic subtypes within each transcriptomic subtype. Relapse-free survival (RFS) was utilized for analysis. **b** RFS of patients with different metabolomic subtypes within BLIS subtype. The *P*-value was calculated using the log rank test. **c** Feature selection with LASSO method for metabolomic subtyping within BLIS tumors. The AUC curve was plotted versus log(λ). Vertical lines were drawn at the optimal values (n = 14 and n = 6) by using the minimum criteria of AUC and the 1 standard error of the minimum criteria (the 1-SE criteria). Six features were selected for the construction of prediction model as they included less metabolites and had relatively high AUC.
