# Supplementary material for: Comprehensive metabolomics expands precision medicine for triple-negative breast cancer
Source: Cell Res. 2022 Feb 1;32(5):477–90. doi: 10.1038/s41422-022-00614-0 (PMC9061756; doi:10.1038/s41422-022-00614-0)
Supplement: Supplementary file 9 — Fig. S8 [file 41422_2022_614_MOESM9_ESM.pdf]

Fig. S8

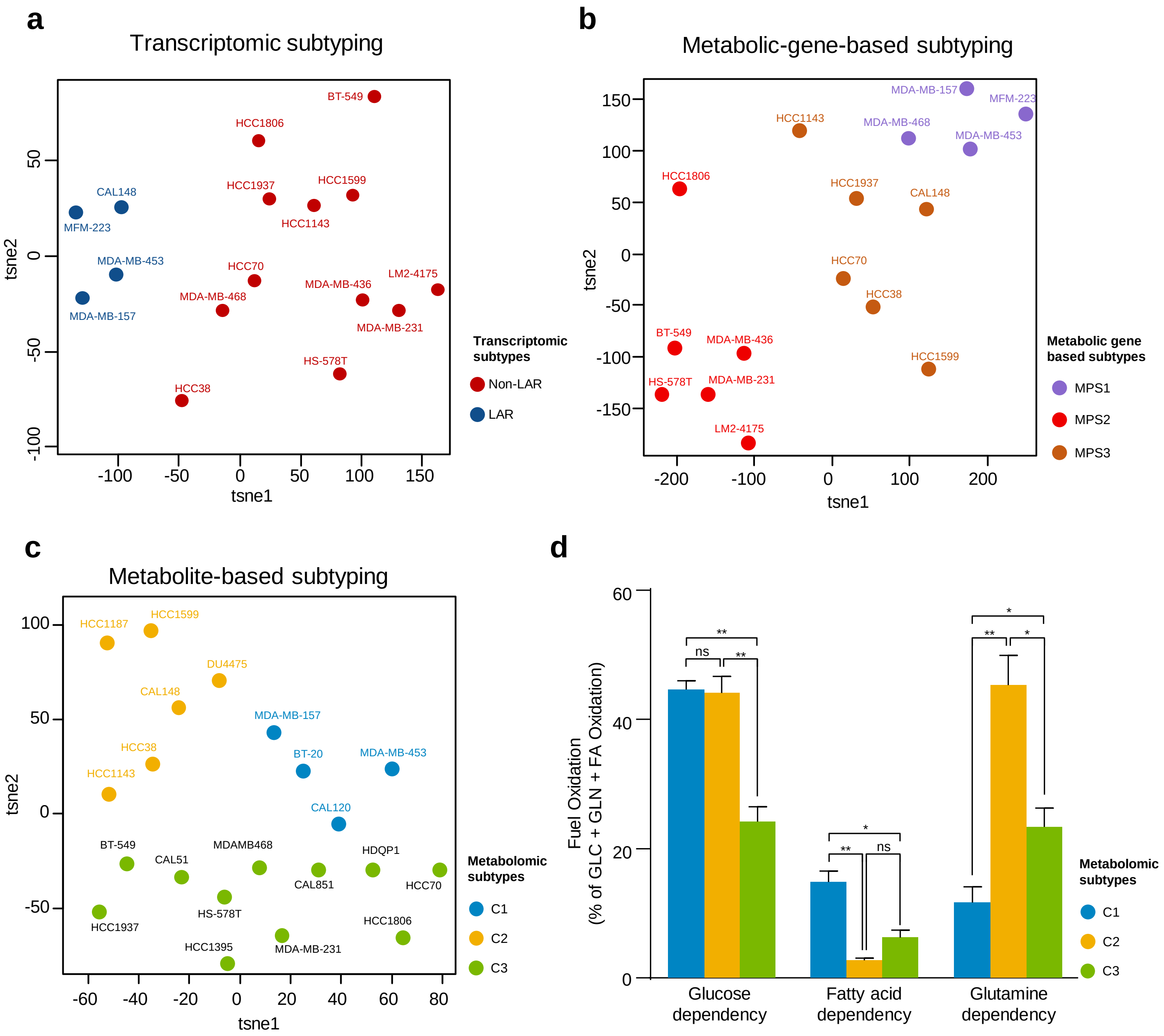

Fig. S8. Subtypes of TNBC cell lines.

a, b Whole transcriptome-based (**a**) and transcriptomic metabolic-gene-based (**b**) subtypings using the RNA-seq data of our cohort. **c** Metabolomic subtyping was based on the metabolomic data of the CCLE dataset. **d** The dependency on each mitochondrial fuel in representative TNBC cell lines of different metabolomic subtypes was detected via seahorse XF mito fuel flex test.. MDA-MB-453, MDA-MB-157 and MFM-223 cell lines (metabolomic C1 subtype); HCC1806, HS-578T and LM2-4175 cell lines (metabolomic C2 subtype); and HCC1143 and CAL148 cell lines (metabolomic C3 subtype) were used for experiments. The fuel oxidation ratio for each cell line was detected with five replicates. Statistical comparisons in **d** were conducted using two-tailed t-test. Data are presented as means ± SEM. *** $P < 0.001$, ** $P < 0.01$; * $P < 0.05$; ns, $P \geq 0.05$.
