# Supplementary material for: Comprehensive metabolomics expands precision medicine for triple-negative breast cancer
Source: Cell Res. 2022 Feb 1;32(5):477–90. doi: 10.1038/s41422-022-00614-0 (PMC9061756; doi:10.1038/s41422-022-00614-0)
Supplement: Supplementary file 10 — Fig. S9 [file 41422_2022_614_MOESM10_ESM.pdf]

Fig. S9

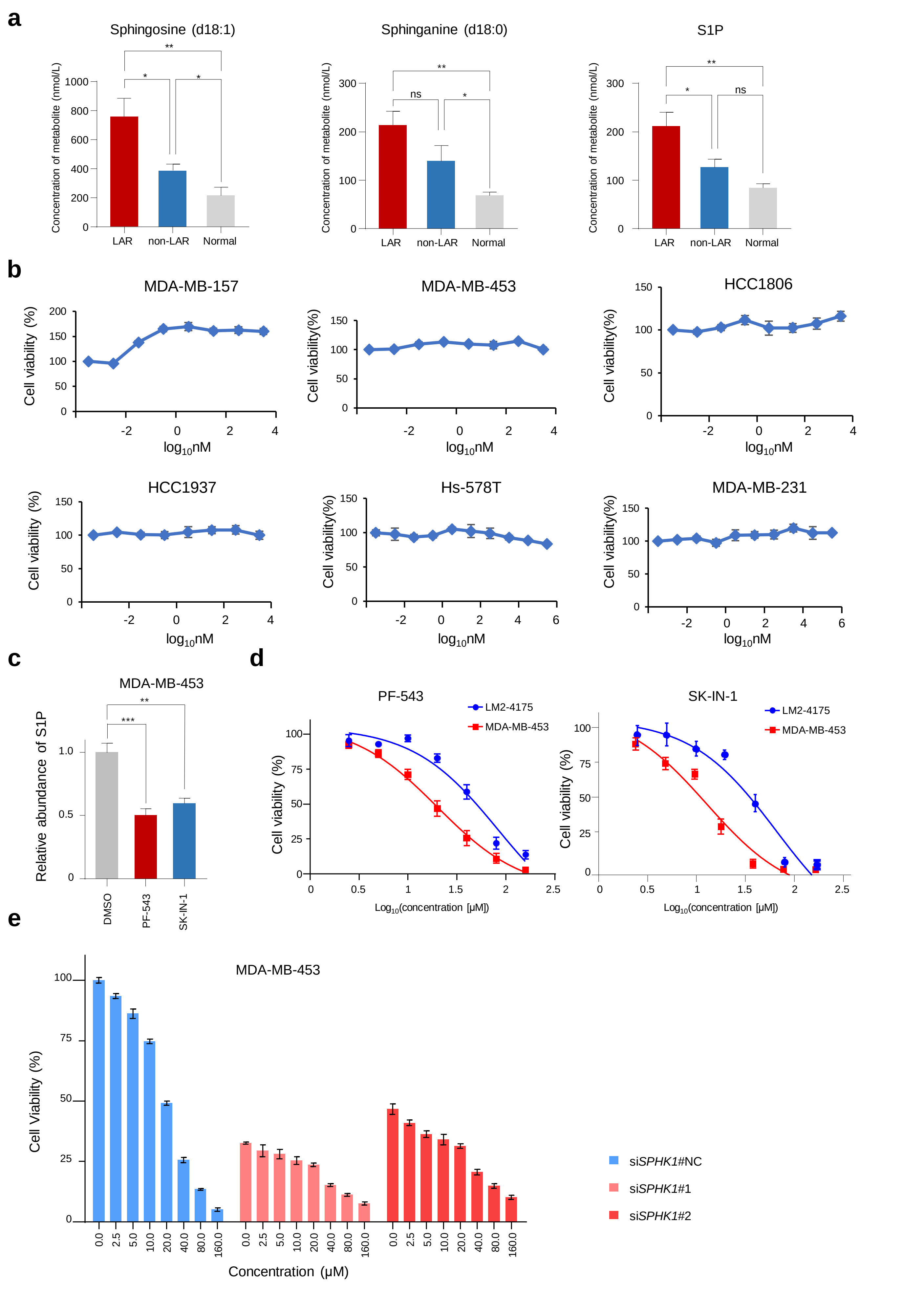
a
Sphingosine (d18:1)
Sphinganine (d18:0)
S1P
Concentration of metabolite (nmol/L)
LAR
non-LAR
Normal
ns
b
MDA-MB-157
MDA-MB-453
HCC1806
HCC1937
Hs-578T
MDA-MB-231
Cell viability (%)
log10nM
c
MDA-MB-453
Relative abundance of S1P
DMSO
PF-543
SK-IN-1
d
PF-543
SK-IN-1
LM2-4175
MDA-MB-453
Cell viability (%)
Log10(concentration [μM])
e
MDA-MB-453
Cell Viability (%)
Concentration (μM)
siSPHK1#NC
siSPHK1#1
siSPHK1#2

Fig. S9. The validation of the crucial role of sphingosine-1-phosphate in LAR tumors.

a Quantification results of the abundances of sphingosine, sphinganine and sphingosine-1-phosphate (S1P) among different transcriptomic subtypes of TNBC samples and normal breast tissues (n=4 for each group). **b** Efficacy of Fumonisin B1 (FB1, the CERS4/6 inhibitor) on the cell variability of different cell lines in short-term (3 days) viability assays. **c** The abundance of S1P upon treatment with control (DMSO), PF-543 (20 μM) or SK-IN-1 (10 μM), two inhibitors of SPHK1, in MDA-MB-453 cells. **d** Efficacy of two SPHK1 inhibitors on LM2-4175 (non-LAR subtype) and MDA-MB-453 (LAR subtype) cell lines. **e** The efficacy of PF-543 after silencing SPHK1 with siRNA on MDA-MB-453 cells. Statistical comparisons in **a** and **c** were conducted using two-tailed t-test. Data are presented as means ± SEM. *** $P < 0.001$, ** $P < 0.01$; * $P < 0.05$; ns, $P \geq 0.05$.
