# Supplementary material for: Comprehensive metabolomics expands precision medicine for triple-negative breast cancer
Source: Cell Res. 2022 Feb 1;32(5):477–90. doi: 10.1038/s41422-022-00614-0 (PMC9061756; doi:10.1038/s41422-022-00614-0)
Supplement: Supplementary file 11 — Fig. S10 [file 41422_2022_614_MOESM11_ESM.pdf]

Fig. S10

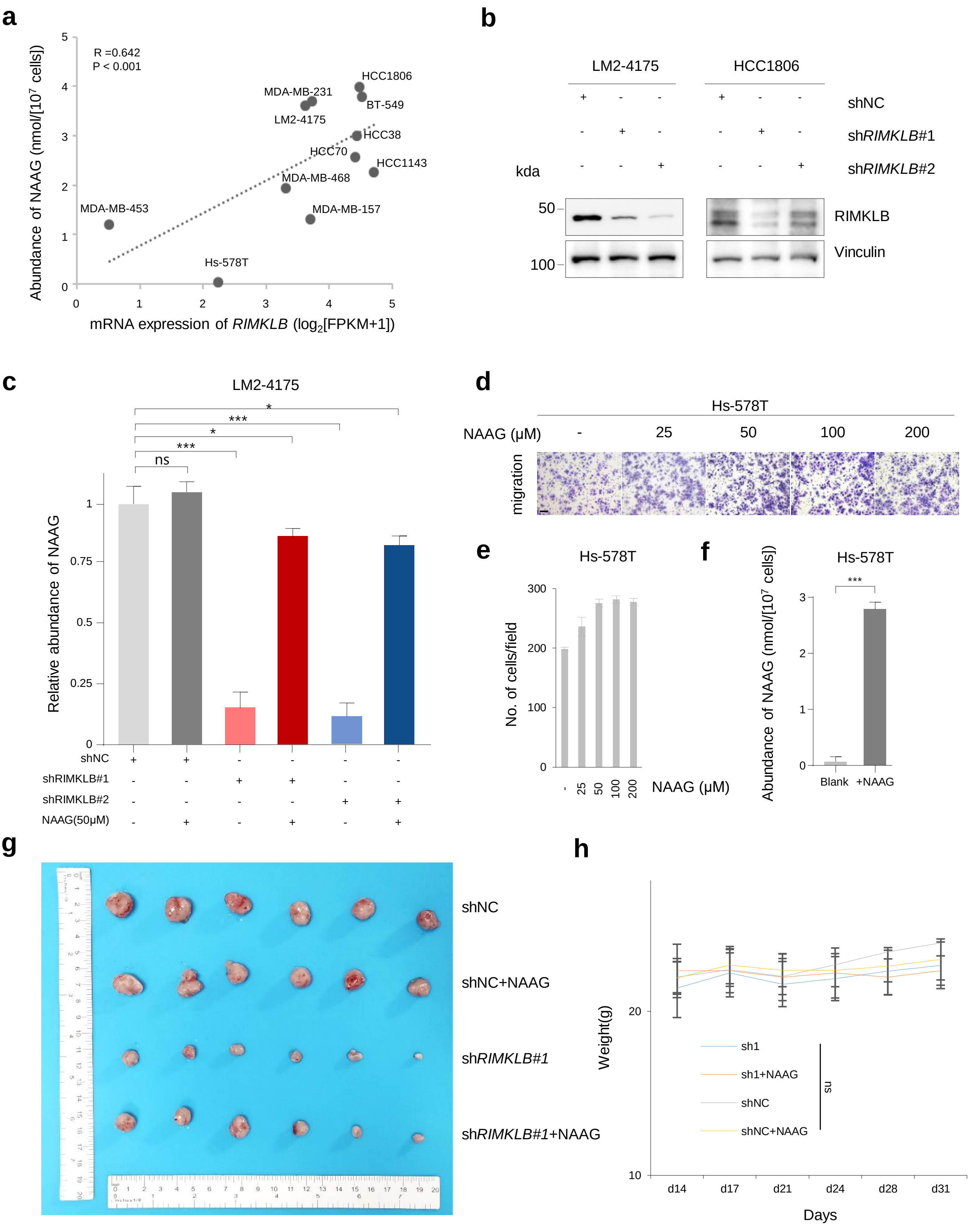
a
R =0.642
P < 0.001
Abundance of NAAG (nmol/[10^7 cells])
mRNA expression of RIMKLB (log2[FPKM+1])
HCC1806
MDA-MB-231
BT-549
LM2-4175
HCC38
HCC70
HCC1143
MDA-MB-468
MDA-MB-453
MDA-MB-157
Hs-578T
b
LM2-4175
HCC1806
shNC
shRIMKLB#1
shRIMKLB#2
kda
50
100
RIMKLB
Vinculin
c
LM2-4175
Relative abundance of NAAG
ns
***
*
shNC
shRIMKLB#1
shRIMKLB#2
NAAG(50μM)
d
Hs-578T
NAAG (μM)
25
50
100
200
migration
e
Hs-578T
No. of cells/field
NAAG (μM)
f
Hs-578T
Abundance of NAAG (nmol/[10^7 cells])
Blank
+NAAG
g
shNC
shNC+NAAG
shRIMKLB#1
shRIMKLB#1+NAAG
h
Weight(g)
sh1
sh1+NAAG
shNC
shNC+NAAG
ns
d14
d17
d21
d24
d28
d31
Days

Fig. S10. Detection of *RIMKLB* expression and investigation of NAAG functions in TNBC cell lines.

a The correlation of *RIMKLB* mRNA expression and NAAG abundance in different TNBC cell lines. **b** Efficacy of *RIMKLB* knockdown in protein level with shRNA in HCC1806 and LM2-4175 cell lines. **c** Effect of *RIMKLB* knockdown and 50 μM NAAG supplement on cell intrinsic NAAG level in LM2-4175 cell line. **d, e** Quantification of cell migrating across transwell filters after adding different concentrations of NAAG in HS-578T cell line. Cells were fixed, stained, visualized at 20× magnification by light microscopy and photographed (**d**). Scale bars, 200 μm. Each panel represents an example of three replicates and ten random fields were counted per insert at 20× (**e**). **f** Effect of 50 μM NAAG supplement into the culture medium on cell intrinsic NAAG level in Hs-578T cells. **g** Pictures of tumors illustrating the effect of *RIMKLB* knockdown and NAAG complement in the *in vivo* experiments. **h** Effect of *RIMKLB* knockdown and NAAG complement on the weight of mice (n=6 for each group). Statistical comparisons in **c** and **f** were conducted using two-tailed t-test. Data are presented as means ± SEM. *** $P < 0.001$, ** $P < 0.01$; * $P < 0.05$; ns, $P \geq 0.05$.
